# Differential Privacy-Based Location and Trajectory Data Protection for Utility-Preserving Location-Based Services

**DOI:** 10.3390/s26175456

**Published:** 2026-08-28

**Authors:** Qihao Yu, Fang Liu, Xianghui Meng, Junjun Ma

**Affiliations:** 1 School of Equipment Engineering, Shenyang Ligong University, Shenyang 110159, China; yuqihao@sylu.edu.cn; 2 School of Information Science and Engineering, Shenyang Ligong University, Shenyang 110158, China; mengxianghui991203@163.com (X.M.); g08852070@gmail.com (J.M.)

**Keywords:** location-based services, location privacy protection, trajectory privacy protection, differential privacy, Q-R tree, spatiotemporal generalization

## Abstract

The widespread use of location-based services (LBSs) has led to the continuous collection of user location and trajectory data, increasing the risk of privacy leakage and creating a persistent tradeoff between privacy protection and data utility. To address this problem in discrete location query scenarios, this paper proposes a single-point location privacy protection method based on Q-R tree retrieval and differential privacy, termed QRDPP. QRDPP combines the adaptive spatial partitioning capability of a Q-tree with the minimum bounding rectangle (MBR)-based indexing capability of an R-tree. It applies an improved geometric privacy budget allocation strategy to leaf nodes and an arithmetic allocation strategy to non-leaf nodes, followed by Laplace perturbation of the corresponding location data and node information. For continuous trajectory query scenarios, this paper proposes a spatiotemporal generalization and differential privacy method, termed STG-DPTP, to address inadequate temporal protection, inappropriate generalization, and trajectory distortion. STG-DPTP performs hierarchical spatiotemporal clustering, separately models temporal and spatial distributions using Gaussian kernel density estimation, dynamically optimizes bandwidth parameters through Bayesian optimization, selects representative candidate subsets using the exponential mechanism, and generates protected trajectories through constrained sampling. Experiments on the GeoLife dataset evaluate the proposed methods in terms of query accuracy, computational efficiency, spatial trajectory similarity, reconstruction error, adversarial uncertainty, and temporal preservation. The results show that QRDPP improves the utility and efficiency of privacy-preserving spatial queries, while STG-DPTP better preserves the spatial distribution, trajectory structure, and temporal characteristics of the original data under the adopted differential privacy framework.

## 1. Introduction

The widespread deployment of wireless communication networks, satellite positioning, and mobile sensing technologies has made location-based services (LBSs) an essential component of modern mobile applications. Services such as navigation, point-of-interest retrieval, vehicle dispatching, location-aware recommendation, and social interaction rely on continuously collected location information to provide timely and personalized responses [1,2,3]. However, the same data that improve service quality can also reveal sensitive personal information. A single location point may disclose a visit to a hospital, workplace, or private residence, while a sequence of locations can expose mobility patterns, daily routines, social relationships, and behavioral preferences.

The privacy risk becomes more serious when location records are accumulated over time. Even when individual records appear isolated or have been partially anonymized, attackers may combine temporal regularity, spatial proximity, and auxiliary background knowledge to reconstruct user identities or movement histories. Existing studies on location inference and trajectory de-anonymization have shown that mobility data remain vulnerable when their spatial and temporal correlations are insufficiently protected [4,5]. Therefore, an effective LBS privacy mechanism must not only conceal individual coordinates, but also preserve service utility and resist inference based on repeated observations.

Current location and trajectory privacy protection methods can be broadly divided into dummy-data generation, generalization and anonymization, suppression, cryptographic protection, and differential privacy [6]. Dummy-based methods conceal a real location or trajectory among artificially generated alternatives. Representative studies have explored online virtual trajectory generation, game-theoretic dummy reporting, and customizable deceptive locations [7,8,9]. These methods are intuitive and compatible with many LBS applications, but their effectiveness depends heavily on whether the generated locations are consistent with realistic mobility patterns. When dummy trajectories exhibit implausible speeds, directions, or semantic behaviors, an attacker may distinguish them from genuine movements.

Generalization and anonymization methods reduce disclosure risk by replacing precise locations with coarser regions or by constructing groups that satisfy anonymity requirements. Existing work has applied dual *k*-anonymity, microaggregation, distributed trust, and credible-chain mechanisms to location or mobility data [10,11,12,13]. Other studies have considered trajectory obfuscation, graph-based access control, and spatial information-loss constraints [14,15,16,17]. Although these approaches can reduce direct identification risk, their performance is sensitive to the selected generalization granularity. Excessive generalization removes useful spatial details, whereas insufficient generalization may retain distinctive mobility patterns.

Suppression-based methods protect privacy by removing sensitive trajectory points, trajectory segments, or local patterns before publication [18,19,20]. Such methods are effective when only a small portion of a trajectory is sensitive, but repeated suppression may damage trajectory continuity and reduce the value of the released data for analysis. Cryptographic approaches provide strong protection during storage, transmission, or secure computation and have been applied to mobile cloud storage, privacy-preserving classification, and encrypted LBS queries [21,22,23]. Nevertheless, cryptographic protection does not directly solve the utility problem of data release, and its computational or communication cost may be unsuitable for large-scale or real-time trajectory processing. Edge- and blockchain-assisted architectures further improve trust management and data integrity [24,25,26], but they still require an effective data-level privacy mechanism when location information is queried or published.

Differential privacy offers a principled alternative by bounding the influence of an individual record on the output of a randomized mechanism [27]. Recent studies have applied differential privacy to location privacy protection in LBSs, location-aware nearest-neighbor queries, taxi-hailing incentives, location-frequency histograms, road-network protection, semantic location protection, and personalized location services [28,29,30,31,32,33]. Other work has investigated trip-oriented data sharing, continuous location protection, three-dimensional geo-indistinguishability, trajectory publishing, and federated spatial range queries [34,35,36,37,38]. These studies demonstrate the flexibility of differential privacy across diverse LBS scenarios. However, they also reveal a persistent tradeoff: stronger perturbation improves privacy but may reduce query accuracy, distort trajectory structure, or weaken temporal consistency. Related physical-layer work has also studied constrained RIS-profile design for OFDM localization; Shi et al. [39] jointly optimize localization accuracy under total-power and unit-modulus constraints. Although that setting differs from mobility-data release, it motivates explicit treatment of coupled constraints, reflected here in privacy-budget conservation and allocation, partition stopping, temporal–spatial composition, and sampling feasibility.

Two limitations are particularly relevant to the scenarios considered in this paper. First, discrete location queries require both efficient spatial retrieval and careful privacy-budget allocation. A simple spatial index may be fast but fail to represent non-uniform location distributions, whereas a hierarchical index may generate excessive nodes and introduce unnecessary perturbation. Moreover, assigning the same privacy budget to different tree levels ignores the distinct roles of leaf and non-leaf nodes. Second, continuous trajectory protection must preserve both spatial distribution and temporal dependence. Methods that protect only coordinates may still expose behavior through timestamps, while independent perturbation of trajectory points can destroy continuity and produce unrealistic paths. Existing trajectory-publishing methods have made progress in data release and continuous-query protection [35,40,41], but balancing temporal privacy, spatial utility, and trajectory realism remains challenging.

Previous studies have shown that the privacy requirements of discrete location queries and continuous trajectory queries are different. For discrete location services, privacy-preserving mechanisms mainly need to protect individual location records while maintaining the accuracy and efficiency of spatial retrieval [28,30]. In contrast, successive location observations and continuous queries introduce additional temporal dependencies and cumulative privacy risks, requiring protection mechanisms that explicitly consider the correlation among consecutive location records [1,35]. Therefore, this study focuses on these two representative query scenarios under the centralized TTP-based LBS architecture.

To address the corresponding privacy challenges, this paper develops two differential privacy-based methods for discrete location queries and continuous trajectory queries, respectively. For discrete location queries, a single-point location privacy protection method based on Q-R tree retrieval and differential privacy, termed QRDPP, is proposed. QRDPP combines the adaptive spatial partitioning capability of a Q-tree with the minimum bounding rectangle (MBR) indexing efficiency of an R-tree. It uses heat density and tree depth as stopping conditions, allocates a total privacy budget across leaf and non-leaf node-count queries, and applies Laplace perturbation to those count statistics. For continuous trajectory queries, a spatiotemporal generalization and differential privacy method, termed STG-DPTP, is proposed. STG-DPTP performs hierarchical temporal and spatial clustering, models the two dimensions using Gaussian kernel density estimation, dynamically optimizes the bandwidth through Bayesian optimization, selects representative candidate subsets through the exponential mechanism, and generates protected trajectories through constrained sampling.

The scope of this study is limited to privacy protection for discrete location queries and continuous trajectory queries under the centralized TTP-based architecture. The proposed methods are not intended to cover all possible LBS privacy scenarios. Other settings, such as decentralized privacy-preserving architectures, user-level trajectory protection, and highly dynamic real-time multi-user environments, are beyond the scope of the present study and will be investigated in future work.

Although differential privacy has been widely applied to LBS privacy protection, existing methods generally focus on specific aspects of location or trajectory privacy. The novelty of this work lies in designing scenario-oriented privacy frameworks according to the intrinsic characteristics of different LBS queries. Specifically, QRDPP jointly considers adaptive spatial organization and differentiated privacy-budget allocation for discrete location queries, while STG-DPTP integrates hierarchical spatiotemporal modeling, adaptive density estimation, and privacy-preserving candidate selection for continuous trajectory queries. Therefore, the proposed methods emphasize the coordinated design of privacy mechanisms rather than the individual adoption of existing techniques.

The main contributions of this paper are summarized as follows. First, QRDPP constructs a hybrid Q-R tree index that improves spatial organization and query efficiency for non-uniform location data while supporting differentiated privacy-budget allocation. Second, STG-DPTP integrates hierarchical spatiotemporal clustering, adaptive density modeling, probabilistic candidate selection, and constrained sampling to protect both spatial and temporal trajectory characteristics. Third, experiments on the GeoLife dataset evaluate the proposed methods in terms of query error, runtime, trajectory similarity, reconstruction error, adversarial uncertainty, and temporal preservation, demonstrating their ability to balance privacy protection and data utility.

### 1.1. System Model and Data Representation

This paper adopts a centralized privacy-preserving architecture for location-based services (LBSs). The architecture contains three participating entities: the user terminal, the trusted third party (TTP) server, and the location-based service provider (LSP). GPS satellites provide positioning signals to the user terminal and are regarded as supporting infrastructure rather than participating entities. A potential attacker is considered an external threat actor and does not participate in the normal service workflow.

**Figure 1:**
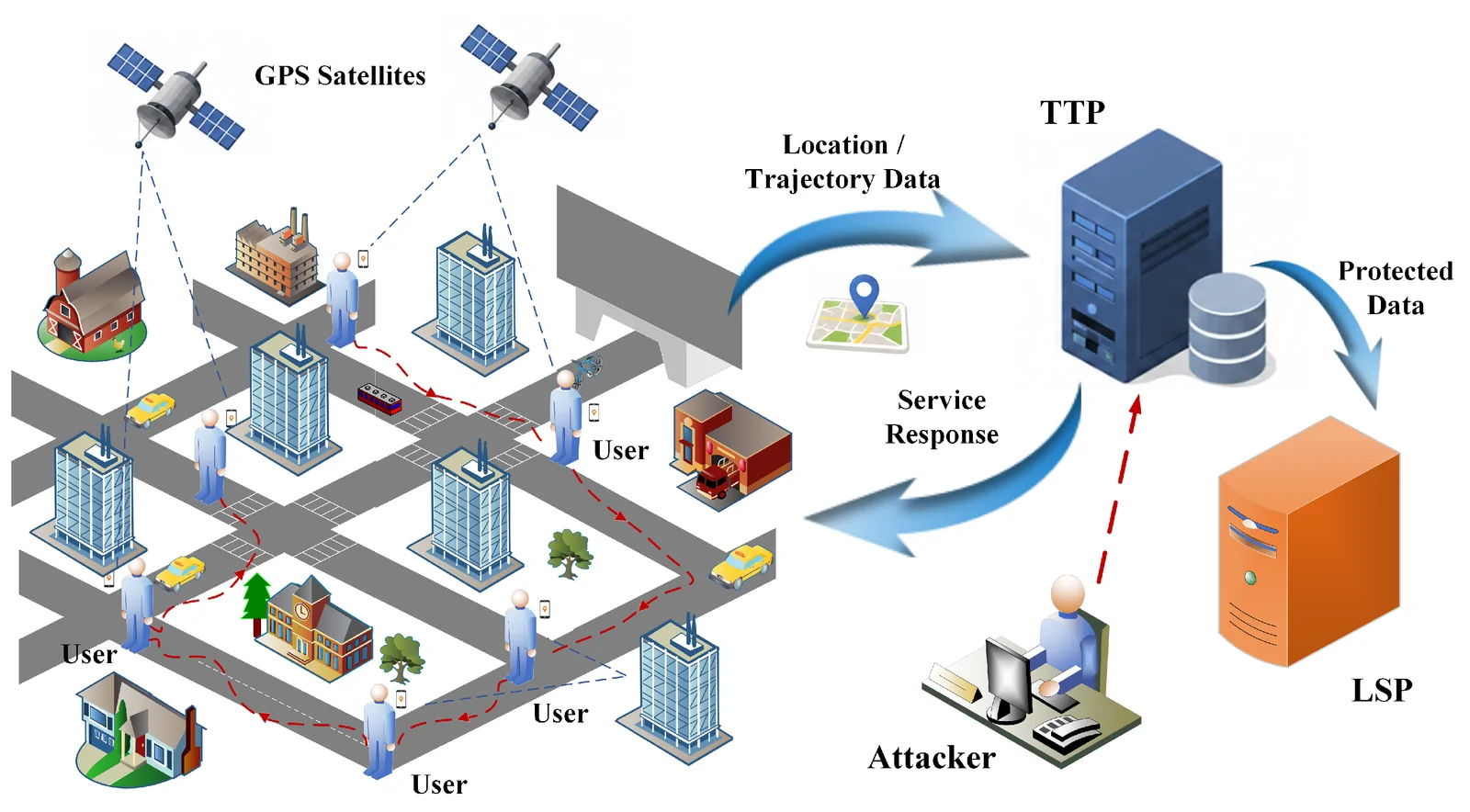
Centralized system model for privacy-preserving location-based services.

As shown in Figure 1, the user terminal obtains location or trajectory data through GPS positioning and submits the original data, together with the corresponding service request, to the TTP through a secure communication channel. According to the temporal continuity of the submitted data, the TTP determines the appropriate privacy-preserving method. For discrete location queries, the TTP applies QRDPP to perform spatial partitioning, privacy-budget allocation, and Laplace perturbation of hierarchical node-count statistics. For continuous trajectory queries, the TTP applies STG-DPTP to perform spatiotemporal clustering, probabilistic modeling, privacy-preserving candidate selection, and constrained sampling.

After privacy-preserving processing, only the noisy query representation produced by QRDPP, or the protected trajectory produced by STG-DPTP, is transmitted to the LSP. The LSP processes the protected request and returns the corresponding service response to the user terminal. Therefore, the user’s original location and trajectory data are accessible only to the trusted TTP and are not directly disclosed to the LSP.

This paper assumes that the TTP is fully trusted and correctly executes the specified privacy-preserving algorithms without disclosing the original data. The LSP is considered an honest-but-curious entity: it follows the prescribed service protocol but may attempt to infer users’ sensitive locations, movement patterns, or activity habits from the protected data by using background knowledge, linkage analysis, or reconstruction attacks. External attackers may also obtain released data and conduct similar inference attacks, but they are assumed to be unable to compromise the TTP or the secure communication channels. Accordingly, the objective of the proposed architecture is to prevent the LSP and external attackers from inferring users’ original location and trajectory information while maintaining the utility of LBS queries and service responses.

The architecture therefore follows the central-curator differential privacy model, and the TTP block in Figure 1 defines the central trust boundary. Secure channels protect data in transit but do not remove the single-point-of-failure risk associated with TTP compromise, insider misuse, software faults, unauthorized storage access, or leakage of mechanism randomness. The formal guarantees in this paper apply to the outputs released after QRDPP or STG-DPTP processing; they do not protect raw records against a TTP component that can directly access those records or the unperturbed internal state. Practical deployments should combine the proposed mechanisms with least-privilege access, encrypted short-lived storage, isolated randomness, tamper-evident auditing, and a per-user privacy-budget ledger. Distributed noise generation, secret sharing, secure aggregation, or multiple non-colluding servers are possible extensions for reducing reliance on a single curator.

In the discrete query scenario, the user’s historical location data are represented as:

(1) $$U=\{L_{1},L_{2},\ldots ,L_{N}\},$$

where the *i*-th location point is:

(2) $$L_{i}=\left(x_{i},y_{i},t_{i}\right).$$

Here, $x_{i}$, $y_{i}$, and $t_{i}$ respectively represent the longitude, latitude, and timestamp of the location point. Although the location points in discrete queries are scattered from each other, an attacker may piece multiple single-point locations into an implicit trajectory through long-term accumulation and correlation analysis, and further infer the user’s activity areas and behavioral patterns.

In the continuous query scenario, the user’s trajectory is represented as follows:

(3) $$T=\{p_{1},p_{2},\ldots ,p_{n}\},$$

where:

(4) $$p_{i}=\left(x_{i},y_{i},t_{i}\right),$$

and all trajectory points are arranged in chronological order. A continuous trajectory simultaneously contains spatial location and temporal information, and the spatiotemporal correlation between trajectory points may expose the user’s movement paths, regular locations, and travel patterns.

Therefore, based on the continuity characteristics of the query data, QRDPP is applied to discrete location query scenarios, while STG-DPTP is applied to continuous trajectory query scenarios with explicit consideration of spatiotemporal correlations.

### 1.2. Differential Privacy

Differential privacy provides privacy protection with mathematical guarantees by limiting the influence of adding or removing a single record on the output distribution of a randomized mechanism [27]. QRDPP uses the Laplace mechanism to perturb node-count query vectors according to the privacy budgets allocated at different tree levels, whereas STG-DPTP uses the exponential mechanism to perform probabilistic selection from temporal and spatial candidate datasets.

#### 1.2.1. Differential Privacy Definition

For QRDPP, two location datasets are regarded as neighboring if they differ by the addition or deletion of one location record. For STG-DPTP, the candidate subsets used in temporal and spatial selection are constructed by removing one trajectory point from the cleaned trajectory dataset; therefore, the privacy protection discussed in this paper is record-level or point-level rather than user-level trajectory privacy. For any neighboring datasets *D* and $D^{\prime }$ and any possible output set *S*, if a randomized mechanism *M* satisfies

(5) $$\mathrm{Pr}\left[M\left(D\right)\in S\right]\leq e^{\epsilon }\mathrm{Pr}\left[M\left(D^{\prime }\right)\in S\right],$$

then *M* is said to satisfy ϵ-differential privacy, where ϵ denotes the privacy budget.

The privacy budget controls the tradeoff between privacy protection and data utility. A smaller ϵ generally introduces stronger random perturbation and provides a higher level of privacy protection, whereas a larger ϵ introduces weaker perturbation and usually provides higher data utility.

For a query function *f*, its global sensitivity is defined as

(6) $$\Delta f=\max_{D,D^{\prime }}{∥f\left(D\right)-f\left(D^{\prime }\right)∥}_{1},$$

where *D* and $D^{\prime }$ are any pair of neighboring datasets. The global sensitivity represents the maximum change in the query result caused by changing one record.

#### 1.2.2. Laplace Mechanism

For a numerical query function f(D), the Laplace mechanism achieves differential privacy by adding calibrated random noise to the true query result [27]. Laplace-based perturbation has also been extended to multidimensional geographical locations [36].

(7) $$M\left(D\right)=f\left(D\right)+\mathrm{Lap}\left(\frac{\Delta f}{\epsilon }\right),$$

where Lap(Δf/ϵ) denotes Laplace noise with scale parameter Δf/ϵ. Its variance is

(8) $$\mathrm{Var}=2{\left(\frac{\Delta f}{\epsilon }\right)}^{2}.$$

Accordingly, under a fixed sensitivity, different privacy budgets generate perturbation noise of different scales. In QRDPP, the total record-level budget is divided between leaf and non-leaf node-count releases and then distributed across the relevant tree levels. The corresponding level-specific budgets determine the scales of the Laplace noise added to the count statistics.

#### 1.2.3. Exponential Mechanism

When the result to be released is a candidate dataset, clustering result, or another non-numerical object, the exponential mechanism can be used for privacy-preserving selection. Let *R* denote the candidate output set, let u(D,r) denote the utility score of candidate result *r* on dataset *D*, and let Δu denote the global sensitivity of the utility function. The probability of selecting candidate result *r* is defined as

(9) $$\mathrm{Pr}\left[M\left(D\right)=r\right]=\frac{\exp\left(\frac{\epsilon u(D,r)}{2\Delta u}\right)}{\sum_{r^{\prime }\in R}\exp\left(\frac{\epsilon u(D,r^{\prime })}{2\Delta u}\right)}.$$

A candidate result with a higher utility score has a greater probability of being selected. Compared with directly and deterministically selecting the optimal result, probabilistic selection reduces the risk of exposing the original data through a deterministic output.

In STG-DPTP, temporal and spatial utility scores are constructed separately according to the density characteristics of the corresponding candidate subsets. The exponential mechanism is then applied to the temporal candidate set and the spatial candidate set, respectively. The temporal and spatial selection stages consume privacy budgets $\epsilon_{t}$ and $\epsilon_{s}$, and the total selection budget is expressed as

(10) $$\epsilon_{\mathrm{STG}-\mathrm{DPTP}}=\epsilon_{t}+\epsilon_{s}=\epsilon .$$

The subsequent Gaussian kernel density estimation, bandwidth optimization, and constrained sampling stages follow the selected temporal and spatial candidate models and do not invoke an additional exponential-mechanism selection budget.

#### 1.2.4. Collusion View and Privacy Bounds

Collusion resistance depends on the information available to the coalition. Let Y=M(D) denote one release that satisfies ϵ-differential privacy under the neighboring-dataset definitions stated above. For any randomized post-processing function $g_{C}$ used by a coalition C, any fixed auxiliary information *z* that does not itself disclose the changed raw record, and any output event *S*, the post-processing property gives

(11) $$\mathrm{Pr}\;\left[g_{C}\left(M\left(D\right),z\right)\in S\right]\leq e^{\epsilon }\mathrm{Pr}\;\left[g_{C}\left(M\left(D^{\prime }\right),z\right)\in S\right].$$

Consequently, an honest-but-curious LSP and external attackers that share or jointly analyze identical copies of the same protected output remain subject to the original ϵ bound; the number of copies does not multiply the privacy budget.

If the coalition combines *r* distinct releases generated from the same privacy unit with budgets $\epsilon_{1},\ldots ,\epsilon_{r}$, basic sequential composition yields

(12) $$\epsilon_{\mathrm{total}}\leq \sum_{j=1}^{r}\epsilon_{j}.$$

For QRDPP, nodes at the same fixed tree level represent disjoint spatial regions, so their count releases use parallel composition within that level. Releases across levels use sequential composition. With the level budgets constrained by Equations (20) and (29), and the pool split in Equation (19), the record-level joint release satisfies

(13) $$\epsilon_{\mathrm{QRDPP}}\leq \epsilon_{L}+\epsilon_{N}=\epsilon .$$

For STG-DPTP, the temporal and spatial exponential-mechanism selections compose as $\epsilon_{t}+\epsilon_{s}=\epsilon$ in Equation (10), provided that their utility functions are calibrated to the stated point-level global sensitivities and the subsequent modeling and sampling stages use only the selected protected outputs. Any later access to raw data would require additional privacy accounting.

The last case can bypass the release mechanism and is therefore outside the current threat model. It must be addressed through system-level controls or a distributed-trust architecture rather than by claiming that output differential privacy protects a compromised raw-data holder.

## 2. Methods

### 2.1. Single-Point Location Privacy Protection Method Based on Q-R Tree Retrieval and Differential Privacy

To address unreasonable privacy budget allocation, low spatial query efficiency, and insufficient data utility in discrete query scenarios, this paper proposes a single-point location privacy protection method based on Q-R tree retrieval and differential privacy, namely Q-R Tree-Based Retrieval and Differential Privacy for Point Location Privacy (QRDPP). The method integrates the hierarchical spatial partitioning capability of the Q-tree with the minimum bounding rectangle (MBR) indexing mechanism of the R-tree and determines the stopping conditions according to regional heat density and tree depth. On this basis, an improved geometric privacy budget allocation strategy is adopted for leaf nodes, while an arithmetic privacy budget allocation strategy is adopted for non-leaf nodes. Finally, the Laplace mechanism perturbs the node-count statistics used to answer spatial range queries; QRDPP does not release directly perturbed longitude–latitude points. Differential privacy has previously been applied to nearest-neighbor retrieval, location-frequency statistics, differentially private spatial grids, and federated spatial range queries [28,31,38,42]. These studies demonstrate the feasibility of privacy-preserving spatial queries, while efficient spatial indexing and level-specific privacy budget allocation remain important for location data with non-uniform distributions.

These components are selected according to the characteristics of discrete spatial queries. The Q-tree provides adaptive partitioning for non-uniform location distributions, while the R-tree organizes the resulting MBRs for efficient range retrieval. The differentiated privacy-budget allocation further reflects the different roles of fine-grained leaf data and aggregated non-leaf information. Thus, the proposed design aims to jointly consider spatial adaptivity, retrieval efficiency, and perturbation utility rather than optimizing these components independently.

QRDPP mainly includes four stages: overall framework and data preprocessing, Q-R tree-based spatial partitioning, differentiated privacy budget allocation, and Laplace perturbation and query response.

#### 2.1.1. Overall Framework and Data Preprocessing

**Figure 2:**
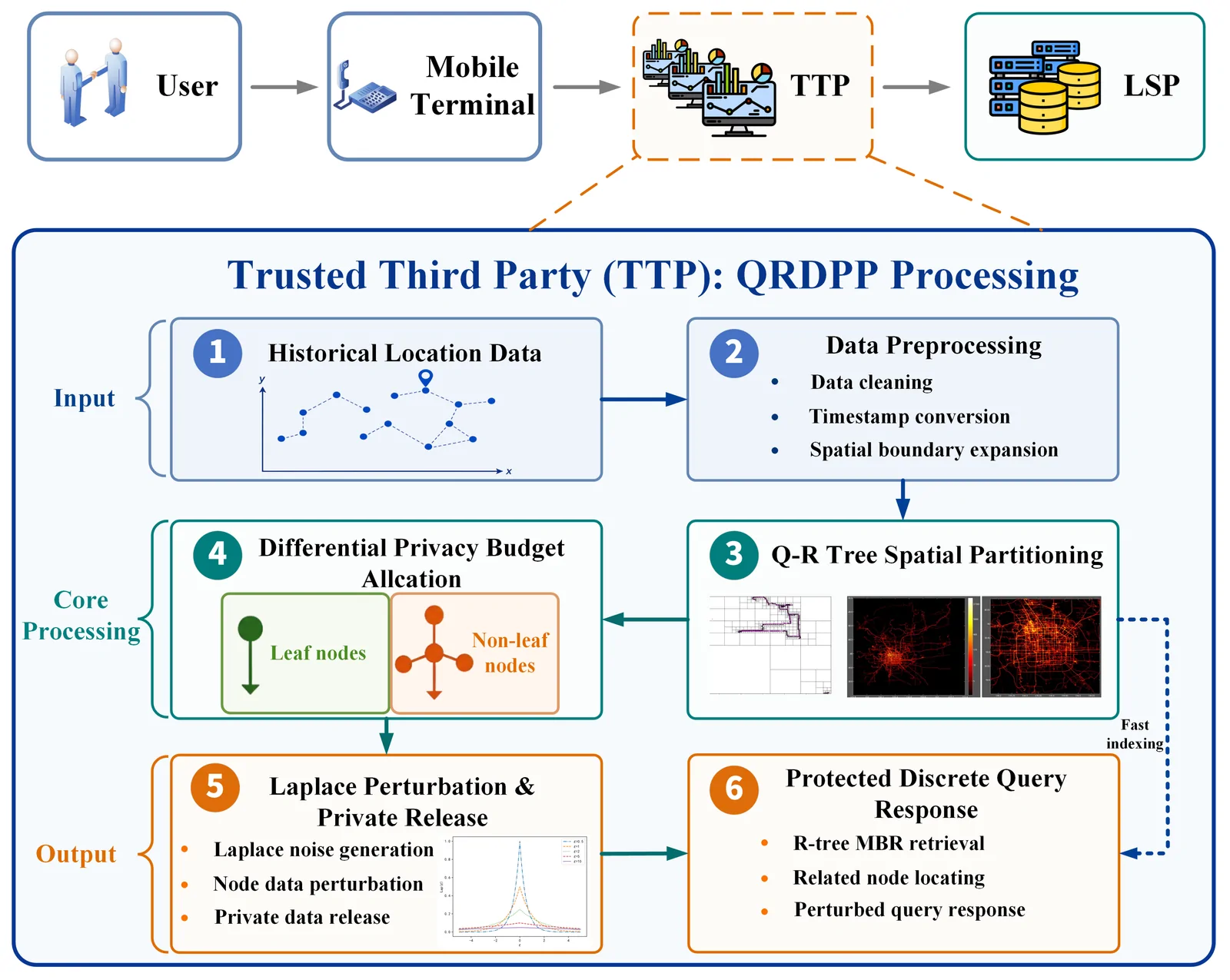
Overall framework and processing workflow of the QRDPP method.

The overall framework and processing workflow of QRDPP are shown in Figure 2. Under the centralized system model described in Section 1.1, the user terminal collects historical location data through GPS positioning and sends the original data to the trusted third party (TTP). The TTP sequentially performs data preprocessing, Q-R tree construction, differentiated privacy-budget allocation, and Laplace perturbation of node counts. Upon receiving a discrete location query request, the TTP uses the established spatial index to locate the relevant nodes and provides only the noisy aggregate query result to the Location-Based Service Provider (LSP).

For a user with several historical location points, let the location dataset be

(14) $$U=\{L_{1},L_{2},\ldots ,L_{i},\ldots ,L_{N}\},$$

where each location point is represented as

(15) $$L_{i}=\left(x_{i},y_{i},t_{i}\right).$$

Here, $x_{i}$, $y_{i}$, and $t_{i}$ denote the longitude, latitude, and timestamp of the *i*-th location point, respectively. The original location data are first cleaned, and the time attribute in the format “YYYY-mm-dd HH:MM:SS” is converted into a timestamp in seconds. The minimum and maximum longitude and latitude values are then used to determine the initial spatial coverage of the quadtree root node.

Because longitude and latitude values are discrete, some boundary points may not be completely covered during recursive partitioning. Therefore, according to the conversion relationship between geographical coordinates and physical distance, the maximum and minimum longitude and latitude bounds of the root node are extended so that all valid location points are included in the initial region. After data cleaning, timestamp conversion, and spatial-boundary extension, the standardized historical location dataset used to construct the Q-R tree is obtained.

#### 2.1.2. Q-R Tree-Based Spatial Partitioning

In the spatial partitioning stage, QRDPP combines the hierarchical spatial segmentation capability of the Q-tree with the MBR indexing mechanism of the R-tree. The Q-tree recursively partitions the two-dimensional space according to the distribution of location points, while the R-tree organizes the MBRs corresponding to the final subregions to support efficient spatial retrieval.

Rectangular regions are adopted because recursive axis-aligned partitioning is naturally compatible with the MBR representation used by the R-tree and enables efficient intersection and range-search operations. This representation serves primarily as a spatial indexing abstraction and does not itself replace or quantize the original GPS coordinates. For irregular geographic objects or road-network regions, an MBR may contain additional space outside the actual geographic shape, which can introduce approximation at region boundaries. Its effect is therefore mainly reflected in partition granularity and candidate retrieval rather than direct coordinate distortion.

For any Q-tree node *v*, let $n_{v}$ denote the number of location points contained in its spatial region, and let $x_{v}^{\min}$, $x_{v}^{\max}$, $y_{v}^{\min}$, and $y_{v}^{\max}$ denote the region boundaries. The area and heat density of node *v* are defined as

(16) $$A_{v}=\left(x_{v}^{\max}-x_{v}^{\min}\right)\left(y_{v}^{\max}-y_{v}^{\min}\right),$$

(17) $$\rho_{v}=\frac{n_{v}}{A_{v}},$$

where $\rho_{v}$ reflects the number of location points per unit coordinate area. Because the experiments use longitude and latitude as the Q-tree coordinates, the numerical value of $\rho_{v}$ is tied to that coordinate scale and should not be interpreted as a physical density in points per square kilometer. The heat density also provides an implicit characterization of the local spatial distribution. Regions containing concentrated location points tend to have larger $\rho_{v}$ and are therefore partitioned more finely, whereas sparse regions terminate earlier and retain coarser partitions. Consequently, the Q-R tree adapts its spatial granularity to non-uniform location distributions, which helps avoid unnecessary subdivision in sparse regions while retaining finer spatial resolution in dense regions. However, density alone does not explicitly model road topology, semantic boundaries, or the internal geometric shape of a point distribution, which remains a limitation of the current partitioning criterion.

Let $d_{v}$ denote the current depth of node *v*, let $d_{\max}$ denote the maximum partitioning depth, and let $\rho_{\mathrm{th}}$ denote the density threshold. The stopping condition is

(18) $$\mathrm{Stop}\left(v\right)\Leftrightarrow \left(d_{v}\geq d_{\max}\right)\vee \left(n_{v}<n_{\min}\right)\vee \left(\rho_{v}<\rho_{\mathrm{th}}\right),$$

where $n_{\min}$ denotes the minimum number of points required for further subdivision. In the experiments, $d_{\max}=11$, $\rho_{\mathrm{th}}=5$, and $n_{\min}=2$. The value $\rho_{\mathrm{th}}=5$ is treated as a preset engineering stopping value and is held fixed in every reported comparison; it was not optimized against the test results. A smaller threshold permits further subdivision of relatively sparse regions, whereas a larger threshold terminates subdivision earlier and reduces tree complexity. Its effect is therefore a direct granularity–complexity tradeoff. Because the numerical density depends on the coordinate scale and data distribution, the adopted threshold is specific to the present implementation and dataset and is not claimed to be universally optimal. Retuning is required when the coordinate system or application region changes.

When the maximum depth is reached, or the regional density is below the threshold, the current node is treated as a leaf node; otherwise, its spatial region is divided into four subregions. The MBRs of the final spatial partitions are then associated with the R-tree to support rapid query-range retrieval.

#### 2.1.3. Differentiated Privacy Budget Allocation

Traditional quadtree-based methods often allocate the same privacy budget to nodes at different levels. However, as the tree extends downward, the spatial regions become finer and the effect of perturbation on data utility changes across levels. In addition, leaf nodes store fine-grained location data, whereas non-leaf nodes mainly store aggregated information and provide index-navigation functions. QRDPP therefore adopts different privacy budget allocation strategies for leaf and non-leaf nodes.

Let ϵ denote the total privacy budget reported for QRDPP. It is divided into a leaf-node pool $\epsilon_{L}$ and a non-leaf-node pool $\epsilon_{N}$ according to

(19) $$\epsilon_{L}=\lambda \epsilon ,\;\epsilon_{N}=\left(1-\lambda \right)\epsilon ,\;\epsilon_{L}+\epsilon_{N}=\epsilon .$$

The reported experiments use the fixed, non-optimized equal split λ=0.5. This closes the privacy accounting without introducing an additional tunable parameter into the comparisons.

(1) Improved geometric budget allocation for leaf nodes

Let *h* denote the maximum depth of the Q-R tree, let $\epsilon_{L}$ denote the budget pool used for leaf nodes, and let $\epsilon_{i}^{L}$ denote the privacy budget assigned to leaf nodes at level *i*. A geometric sequence with ratio *q* is used so that deeper levels receive progressively larger privacy budgets. The total leaf-node budget satisfies

(20) $$\sum_{i=1}^{h}\epsilon_{i}^{L}=\epsilon_{L}.$$

Accordingly, the first-level leaf budget is

(21) $$\epsilon_{1}^{L}=\left\{\begin{array}{cc} \epsilon_{L}\frac{1-q}{1-q^{h}}, & q\neq 1,\\ \frac{\epsilon_{L}}{h}, & q=1, \end{array}\right.$$

and the budget allocated to level *i* is

(22) $$\epsilon_{i}^{L}=\left\{\begin{array}{cc} \epsilon_{L}\frac{\left(1-q\right)q^{i-1}}{1-q^{h}}, & q\neq 1,\\ \frac{\epsilon_{L}}{h}, & q=1, \end{array}\right.\;i=1,2,\ldots ,h.$$

Let $n_{i}$ denote the number of leaf nodes at level *i*. Under the Laplace mechanism, the accumulated perturbation error at that level is represented as

(23) $$E\left(Q_{i}\right)=\frac{2n_{i}{\left(\Delta f\right)}^{2}}{{\left(\epsilon_{i}^{L}\right)}^{2}}.$$

Here, $E(Q_{i})$ denotes the aggregate expected squared perturbation error of the leaf-node count statistics at level *i*, rather than the empirical range-query error reported in the experiments. Each leaf-node count is perturbed by independent zero-mean Laplace noise with variance $2{\left(\Delta f/\epsilon_{i}^{L}\right)}^{2}$. Therefore, for $n_{i}$ leaf nodes at level *i*, the accumulated error is obtained by summing these individual variances. Under the independence assumption, the total theoretical perturbation error over all leaf levels is $E\left(Q\right)=\sum_{i}E\left(Q_{i}\right)$.

The total error over all leaf levels is

(24) $$E\left(Q\right)=\sum_{i=1}^{h}E\left(Q_{i}\right)=\sum_{i=1}^{h}\frac{2n_{i}{\left(\Delta f\right)}^{2}}{{\left(\epsilon_{i}^{L}\right)}^{2}}.$$

The ratio can be obtained from the stated error objective rather than from monotonicity alone. Minimizing Equation (24) subject to Equation (20) gives the Lagrangian

(25) $$L=\sum_{i=1}^{h}\frac{2n_{i}{\left(\Delta f\right)}^{2}}{{\left(\epsilon_{i}^{L}\right)}^{2}}+\mu \left(\sum_{i=1}^{h}\epsilon_{i}^{L}-\epsilon_{L}\right).$$

The first-order condition implies ${\left(\epsilon_{i}^{L}\right)}^{3}\propto n_{i}$, and hence

(26) $$\frac{\epsilon_{i+1}^{L}}{\epsilon_{i}^{L}}={\left(\frac{n_{i+1}}{n_{i}}\right)}^{1/3}.$$

The fixed-ratio implementation adopts the design approximation $n_{i+1}/n_{i}\approx 2$, reflecting that sparse quadtree branches terminate before all four children remain active. Under this approximation, the geometric ratio is

(27) $$q=\sqrt[{3}]{2}.$$

Thus, deeper leaf levels receive larger privacy budgets and correspondingly smaller perturbation scales. Since $q=\sqrt[{3}]{2}>1$, the allocation satisfies $\epsilon_{i+1}^{L}=q\epsilon_{i}^{L}>\epsilon_{i}^{L}$. The doubling assumption is a budget-design heuristic, not a universal property of every data-dependent Q-tree; an implementation that estimates the active-node ratio at each level could instead use Equation (26) adaptively.

(2) Arithmetic budget allocation for non-leaf nodes

Non-leaf nodes mainly store aggregated information and provide hierarchical navigation for spatial queries. Let $\epsilon_{N}$ denote the budget pool used for non-leaf nodes, let $\epsilon_{j}^{N}$ denote the privacy budget assigned to level *j*, and let *d* denote the arithmetic adjustment coefficient. The non-leaf budget is defined as

(28) $$\epsilon_{j}^{N}=\frac{\epsilon_{N}}{h+1}+\left(j-\frac{h}{2}\right)d,\;0\leq j\leq h.$$

These level budgets satisfy

(29) $$\sum_{j=0}^{h}\epsilon_{j}^{N}=\epsilon_{N}.$$

When d≥0, the smallest budget occurs at level j=0. To ensure that all level budgets remain positive, *d* is selected to satisfy

(30) $$0\leq d<\frac{2\epsilon_{N}}{h(h+1)}.$$

Through the geometric and arithmetic allocation strategies, QRDPP assigns different perturbation scales to fine-grained leaf-node data and aggregated non-leaf-node information. Equations (20), (29), and (19) ensure that the component budgets compose to the reported total budget ϵ.

#### 2.1.4. Laplace Perturbation and Query Response

After the privacy budgets have been allocated, QRDPP applies the Laplace mechanism to the count statistics associated with the Q-R tree nodes. For a node *v*, let $c_{v}\left(D\right)$ denote the number of location records contained in the spatial region represented by node *v*. The corresponding query function is defined as

(31) $$f_{v}\left(D\right)=c_{v}\left(D\right).$$

Under the record-level neighboring relation adopted in this study, two datasets *D* and $D^{\prime }$ differ by the addition or removal of one location record. Therefore, the count of any individual node can change by at most one, and the global sensitivity of the node-count query is

(32) $$\Delta f_{v}=\max_{D∼D^{\prime }}\left|c_{v}\left(D\right)-c_{v}\left(D^{\prime }\right)\right|=1.$$

For a fixed spatial hierarchy, the regions at any one level are disjoint. Consequently, adding or removing one record changes one component of the level count vector by one, so its $\ell_{1}$ sensitivity is also 1. The same record can affect one node at each non-leaf level, and sequential composition is therefore applied across those levels. The leaf counts constitute disjoint terminal regions; using the conservative allocation in Equation (20), their release consumes no more than $\epsilon_{L}$. The non-leaf releases consume $\sum_{j}\epsilon_{j}^{N}=\epsilon_{N}$. Releasing both collections thus consumes no more than $\epsilon_{L}+\epsilon_{N}=\epsilon$ under Equation (19).

Thus, the sensitivity parameter used by QRDPP is Δf=1. For a leaf node located at level *i*, the privacy budget $\epsilon_{i}^{L}$ determined by the leaf-node allocation strategy is used to generate independent Laplace noise:

(33) $$\eta_{v}^{L}∼\mathrm{Lap}\left(\frac{\Delta f}{\epsilon_{i}^{L}}\right),\;\Delta f=1.$$

The privacy-protected count of the leaf node is therefore

(34) $${\tilde{c}}_{v}=c_{v}+\eta_{v}^{L}.$$

Similarly, for a non-leaf node *v* at level *j*, the privacy budget $\epsilon_{j}^{N}$ determined by the arithmetic allocation strategy is used. The corresponding perturbation is

(35) $$\eta_{v}^{N}∼\mathrm{Lap}\left(\frac{\Delta f}{\epsilon_{j}^{N}}\right),\;{\tilde{c}}_{v}=c_{v}+\eta_{v}^{N}.$$

Therefore, QRDPP perturbs the node-count statistics rather than directly modifying the original longitude and latitude coordinates. The spatial coordinates are used internally by the TTP to construct the Q-R tree and determine the spatial regions associated with individual nodes, while the privacy-preserving query response is generated from the perturbed node statistics.

The stated record-level guarantee is conditional on treating the spatial domain and hierarchy as fixed for a release epoch and on not publishing the unperturbed tree structure. If a data-dependent partition itself is released or rebuilt as part of every neighboring input, its construction requires a separate privacy analysis or a private partitioning mechanism. That extension is outside the scope of the current evaluation.

Upon receiving a discrete range-query request, the TTP first uses the R-tree-based MBR index to identify the Q-tree nodes whose spatial regions intersect the query range. If a node region is fully covered by the query range, its perturbed count ${\tilde{c}}_{v}$ is directly used in the query response. For a leaf node that is only partially intersected by the query range, its perturbed count is weighted according to the overlap ratio between the query rectangle and the node MBR. Let $R_{q}$ denote the query rectangle and $R_{v}$ denote the MBR of leaf node *v*. The overlap ratio is defined as

(36) $$\alpha \left(R_{q},R_{v}\right)=\frac{\mathrm{Area}(R_{q}\cap R_{v})}{\mathrm{Area}(R_{v})},\;0\leq \alpha \left(R_{q},R_{v}\right)\leq 1.$$

Accordingly, the contribution of a partially intersected leaf node to the query result is estimated as

(37) $${\hat{c}}_{v}=\alpha \left(R_{q},R_{v}\right){\tilde{c}}_{v}.$$

The final privacy-preserving range-query result is obtained by aggregating the perturbed counts of the fully covered nodes and the weighted perturbed counts of partially intersected leaf nodes. Since this query-response procedure operates only on the already perturbed node statistics, the subsequent MBR retrieval, overlap calculation, and aggregation are post-processing operations and do not require additional access to the original location coordinates.

In this way, QRDPP combines Q-R tree-based spatial retrieval, differentiated privacy-budget allocation, and Laplace perturbation of node-count statistics. The sensitivity Δf=1 has a clear interpretation under the adopted record-level neighboring relation, while the level-specific privacy budgets control the corresponding Laplace noise scales and the resulting privacy–utility tradeoff.

### 2.2. Trajectory Privacy Protection Method Based on Spatiotemporal Generalization and Differential Privacy

To address the problems of significant spatiotemporal correlation in trajectory data under continuous query scenarios, improper generalization by traditional methods, and the neglect of privacy risks in the temporal dimension due to protecting only the spatial dimension, this paper proposes a trajectory privacy protection method based on spatiotemporal generalization and differential privacy (spatiotemporal generalization with differential privacy for trajectory protection, STG-DPTP). This method utilizes the hierarchical clustering capability of Bisecting K-means to subdivide the temporal and spatial features of trajectory data, adopts Gaussian kernel density estimation to fit the temporal and spatial data distributions respectively, combines Bayesian optimization to dynamically determine model parameters, and uses the exponential mechanism to make probabilistic selections from candidate datasets, ultimately generating perturbed trajectory data through constrained sampling.

The components of STG-DPTP are selected to address different aspects of continuous trajectory protection: hierarchical clustering captures heterogeneous spatiotemporal patterns, KDE models local data distributions without assuming a fixed parametric form, Bayesian optimization adapts the bandwidth to different clusters, the exponential mechanism provides private candidate selection, and constrained sampling helps preserve trajectory continuity.

STG-DPTP mainly consists of four stages: overall framework and trajectory preprocessing, hierarchical spatiotemporal clustering, privacy-preserving spatiotemporal modeling and candidate selection, and constrained trajectory generation and publication.

#### 2.2.1. Overall Framework and Trajectory Preprocessing

**Figure 3:**
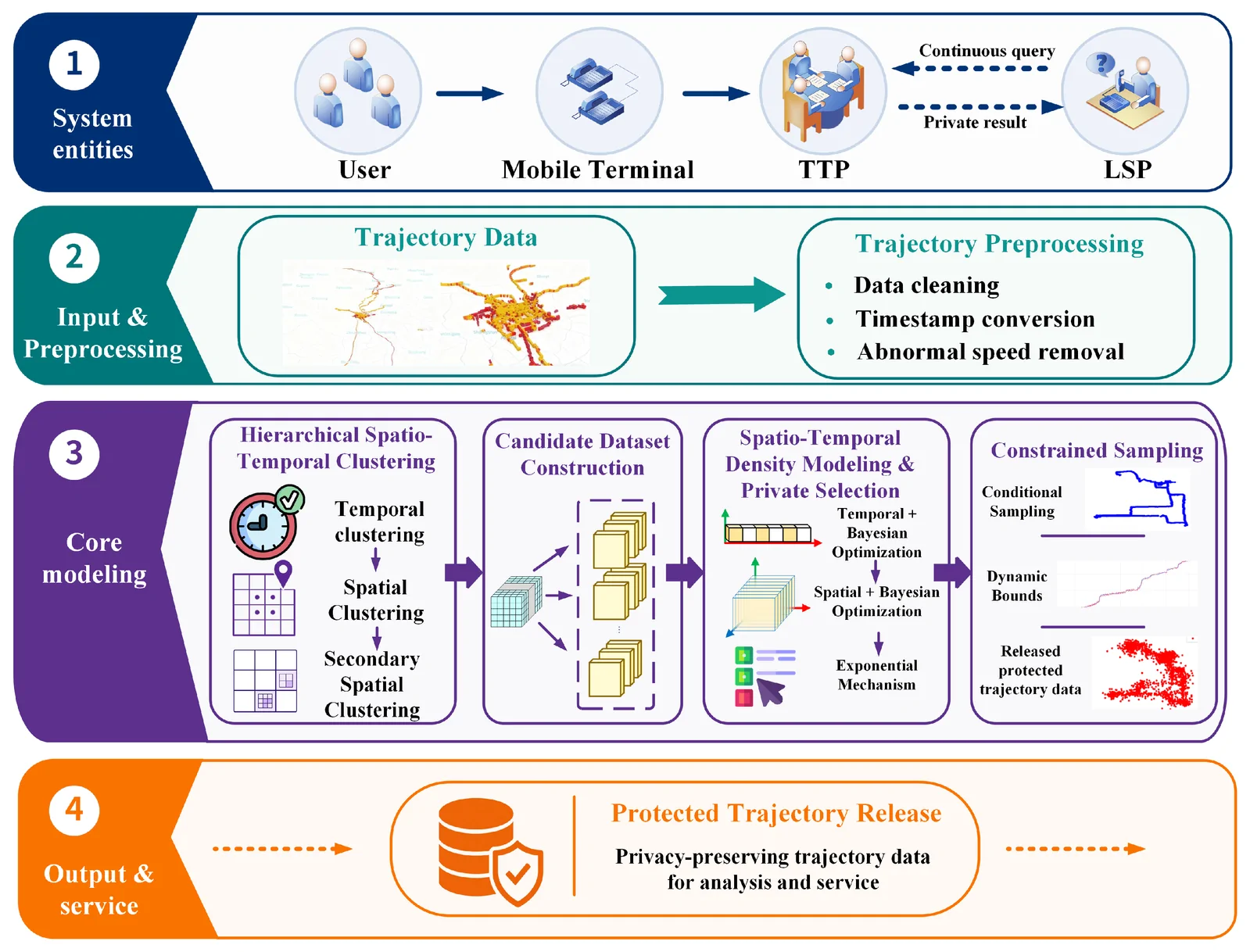
Overall framework and processing workflow of the STG-DPTP method.

The overall processing workflow of STG-DPTP is shown in Figure 3. Under the centralized system architecture described in Section 1.1, the user terminal obtains mobile trajectory data containing spatial coordinates and timestamp information through GPS positioning technology, and sends the original trajectory to the trusted third party (TTP) server. The TTP utilizes STG-DPTP to complete the privacy-preserving processing of trajectory data, and then sends the processed trajectory data to the location service provider (LSP), which responds to the user’s continuous query requests based on the protected data and returns the service results. Previous studies have shown that successive location records exhibit strong dependency and that continuous queries require dedicated privacy mechanisms rather than independent point-wise protection [1,35].

Let the original trajectory dataset to be protected be:

(38) $$T=\{p_{1},p_{2},\ldots ,p_{n}\},$$

where each trajectory point is represented as follows:

(39) $$p_{i}=\left(x_{i},y_{i},t_{i}\right),$$

where $x_{i},y_{i}$, and $t_{i}$ respectively represent the longitude, latitude, and timestamp of the *i*-th trajectory point. The original trajectory points are first sorted in ascending order of their timestamps, and records with missing coordinates, invalid timestamps, or duplicated timestamps are removed. The time attribute in the format “YYYY-mm-dd HH:MM:SS” is converted into seconds. Since longitude and latitude are angular coordinates, the geographical distance between adjacent trajectory points is calculated using the Haversine formula rather than directly applying the Euclidean distance to the longitude–latitude values. Let $\lambda_{i}=\pi x_{i}/180$ and $\phi_{i}=\pi y_{i}/180$ denote the longitude and latitude of the *i*-th trajectory point in radians, respectively. For i=2,3,…,n, define

(40) $$a_{i}=\sin^{2}\left(\frac{\phi_{i}-\phi_{i-1}}{2}\right)+\cos\phi_{i-1}\cos\phi_{i}\sin^{2}\left(\frac{\lambda_{i}-\lambda_{i-1}}{2}\right),$$

and the geographical distance between two adjacent points is

(41) $$D_{i-1,i}=2R_{E}atan2\left(\sqrt{a_{i}},\sqrt{1-a_{i}}\right),\;i=2,3,\ldots ,n,$$

where $R_{E}=6371.0088\;\mathrm{km}$ is the mean radius of the Earth. Since duplicated and non-increasing timestamps have been removed, $t_{i}-t_{i-1}>0$ holds for all retained adjacent points. The average moving speed, measured in km/h, is defined as

(42) $$v_{i}=\left\{\begin{array}{cc} 0, & i=1,\\ \frac{3600D_{i-1,i}}{t_{i}-t_{i-1}}, & 2\leq i\leq n. \end{array}\right.$$

A trajectory point is regarded as an abnormal point when its associated speed exceeds a predefined threshold $v_{\max}$. After removing the abnormal points and reordering the remaining records chronologically, the cleaned trajectory dataset is denoted by

(43) $$T^{\prime }=\left\{p_{1}^{\prime },p_{2}^{\prime },\ldots ,p_{m}^{\prime }\right\},\;m\leq n.$$

The cleaned dataset is subsequently used for temporal clustering, spatial clustering, privacy-preserving candidate selection, and trajectory generation.

#### 2.2.2. Hierarchical Spatiotemporal Clustering

In the spatiotemporal clustering stage, STG-DPTP adopts Bisecting K-means to hierarchically partition the temporal dimension and spatial dimension of the trajectory data. This algorithm inherits the characteristics of traditional K-means, being simple, efficient, and suitable for large-scale datasets, while reducing sensitivity to initial centroids through an iterative bisection strategy, decreasing the risk of falling into local optimal solutions, and making the clustering results more stable.

In this paper, Bisecting K-means is used to minimize the sum of squared errors within clusters and hierarchically partition the temporal and spatial dimensions of trajectory data. This section focuses on explaining the spatiotemporal hierarchical application process of this algorithm in STG-DPTP.

For temporal clustering, each timestamp is first converted into the corresponding local time, and its seconds within a day are calculated as

(44) $$\tau_{i}=3600H_{i}+60M_{i}+S_{i},\;0\leq \tau_{i}<86400,$$

where $H_{i}$, $M_{i}$, and $S_{i}$ denote the hour, minute, and second components of timestamp $t_{i}$, respectively. The temporal feature set is therefore represented as

(45) $$D_{\mathrm{time}}=\left\{\tau_{1},\tau_{2},\ldots ,\tau_{m}\right\}.$$

Bisecting K-means is then applied to $D_{\mathrm{time}}$ to group trajectory records with similar times of day. Let $K_{t}$ denote the number of temporal clusters. In this study, $K_{t}$ is set to 48 to obtain an average temporal granularity approximately comparable to 30 min over a 24-h period. However, because Bisecting K-means performs data-driven partitioning, the resulting temporal clusters are not necessarily equal in duration. According to the clustering results, the cleaned trajectory data are divided into

(46) $$T_{\mathrm{time}}=\left\{T_{1}^{\left(t\right)},T_{2}^{\left(t\right)},\ldots ,T_{K_{t}}^{\left(t\right)}\right\},\;K_{t}=48.$$

Each temporal cluster contains the labels and time-of-day values of its associated trajectory records. The clusters are ordered according to their centroid times, and the record counts and unique labels are checked to ensure that all cleaned trajectory points are assigned exactly once.

Subsequently, extract the corresponding position points based on the labels in each sub-temporal dataset to form a spatial position point set:

(47) $$D_{location}=\left\{\left(x_{1},y_{1}\right),\left(x_{2},y_{2}\right),\ldots ,\left(x_{i},y_{i}\right),\ldots ,\left(x_{n},y_{n}\right)\right\}.$$

In order to maintain the consistency of the clustering method, the spatial dimension is still partitioned using Bisecting K-means, obtaining sub-position datasets corresponding to the 48 sub-temporal datasets:

(48) $$Location\_s=\{P_{1},P_{2},\ldots ,P_{48}\}.$$

Each spatial cluster represents a geographical aggregation area of trajectory points within the corresponding time period, which can reflect the user’s typical activity range during that period.

To maintain temporal consistency and to further mine fine-grained activity hotspots in the spatial dimension and retain the spatial distribution characteristics of the original trajectory at a finer scale, secondary Bisecting K-means clustering is continued on the sub-position datasets to obtain the grandchild position datasets:

(49) $$Location\_g=\{P_{1},P_{2},\ldots ,P_{48K_{2}}\},$$

where $K_{2}$ represents the preset number of clusters for the secondary spatial clustering. Through temporal clustering, spatial clustering, and secondary spatial clustering, the original trajectory data are organized into hierarchical spatiotemporal sub-datasets with temporal consistency and fine-grained spatial features, providing a foundation for subsequent probabilistic modeling and data sampling.

#### 2.2.3. Privacy-Preserving Spatiotemporal Modeling and Candidate Selection

The clustering results may contain information related to the original trajectory distribution. If these intermediate results are directly released, an attacker may infer sensitive trajectory characteristics from the clustering structure. To reduce the leakage risk associated with deterministic publication, STG-DPTP applies Gaussian kernel density estimation to the temporal and spatial dimensions separately, dynamically optimizes the bandwidth parameters through Bayesian optimization, and uses the exponential mechanism to select representative temporal and spatial candidate subsets. Differential privacy has been applied to trajectory publication, trip-oriented data sharing, and hierarchical trajectory storage, demonstrating that probabilistic perturbation can protect trajectory records while retaining aggregate mobility characteristics [34,37,40].

For the cleaned trajectory dataset

(50) $$T^{\prime }=\left\{p_{1}^{\prime },p_{2}^{\prime },\ldots ,p_{m}^{\prime }\right\},$$

the *i*-th leave-one-out candidate subset is defined as

(51) $$C_{i}=T^{\prime }\setminus \left\{p_{i}^{\prime }\right\},\;1\leq i\leq m,$$

and the candidate collection is

(52) $$\Phi_{\mathrm{traj}}=\left\{C_{1},C_{2},\ldots ,C_{m}\right\}.$$

Each candidate subset contains m−1 trajectory points and is used for subsequent temporal and spatial density modeling. The notation $C_{i}$ is used here to distinguish the candidate subsets from the spatial clusters $P_{i}$ defined in the preceding subsection.

Gaussian kernel density estimation is then used to fit the temporal and spatial distributions of the candidate subsets, and Bayesian optimization is employed to dynamically adjust the bandwidth parameters. For each temporal candidate subset, one-dimensional Gaussian kernel density estimation is used to fit the timestamp distribution. The empirical bandwidth score is defined as

(53) $$score\left(h\right)=-\frac{1}{q}\sum_{j=1}^{q}\left|{\hat{x}}_{j}-x_{j}\right|,$$

where *q* is the number of samples used for bandwidth evaluation, ${\hat{x}}_{j}$ is the *j*-th sample generated using bandwidth *h*, and $x_{j}$ is the corresponding evaluation sample. A larger value of score(h) indicates a smaller empirical reconstruction error and therefore a better bandwidth under the adopted evaluation criterion.

After temporal density fitting, the weighted temporal density score of candidate subset $C_{i}$ is calculated as

(54) $$score_{\mathrm{time}}\left(i\right)=\frac{\sum_{k}w_{k}d_{k}}{\sum_{k}w_{k}},$$

where $w_{k}$ denotes the number of trajectory points in the *k*-th temporal cluster and $d_{k}$ denotes the temporal density score obtained from the fitted temporal model. According to the temporal privacy budget $\epsilon_{t}$, the utility sensitivity Δu, and the temporal density score, the exponential mechanism selects a temporal candidate according to

(55) $$\mathrm{Pr}\left[M_{t}\left(D\right)=i\right]=\frac{\exp\left(\frac{\epsilon_{t}\;score_{\mathrm{time}}\left(i\right)}{2\Delta u}\right)}{\sum_{r\in R_{t}}\exp\left(\frac{\epsilon_{t}\;score_{\mathrm{time}}\left(r\right)}{2\Delta u}\right)},$$

where $R_{t}$ denotes the temporal candidate output set. A candidate subset with a higher temporal density score has a greater probability of being selected.

For the grandchild spatial datasets obtained by secondary clustering, two-dimensional Gaussian kernel density estimation is used to fit the coordinate distribution. Bayesian optimization based on the same empirical bandwidth criterion is used to determine the longitude and latitude bandwidth parameters. The weighted spatial density score of candidate subset $C_{i}$ is defined as

(56) $$score_{\mathrm{space}}\left(i\right)=\frac{\sum_{k}w_{k}s_{k}}{\sum_{k}w_{k}},\;s_{k}=\frac{n_{k}}{{\bar{d}}_{k}},$$

where $w_{k}$ denotes the number of data points in the *k*-th spatial cluster, $n_{k}$ denotes the number of points in that cluster, ${\bar{d}}_{k}$ denotes the average Euclidean distance from the points in the cluster to the cluster center, and $s_{k}$ denotes the spatial density score. According to the spatial privacy budget $\epsilon_{s}$, the spatial candidate is selected with probability

(57) $$\mathrm{Pr}\left[M_{s}\left(D\right)=i\right]=\frac{\exp\left(\frac{\epsilon_{s}\;score_{\mathrm{space}}\left(i\right)}{2\Delta u}\right)}{\sum_{r\in R_{s}}\exp\left(\frac{\epsilon_{s}\;score_{\mathrm{space}}\left(r\right)}{2\Delta u}\right)},$$

where $R_{s}$ denotes the spatial candidate output set.

Through separate probabilistic selection in the temporal and spatial dimensions, STG-DPTP obtains candidate models with high temporal representativeness and spatial distribution quality. These selected models are then used as the basis for the original dynamic-bandwidth KDE fitting and constrained trajectory sampling procedure evaluated in the experiments.

#### 2.2.4. Constrained Trajectory Generation and Publication

In the data-constrained sampling stage, STG-DPTP generates perturbed trajectory data based on the optimal temporal candidate set and spatial candidate set filtered out by the exponential mechanism. The sampling process not only generates new samples based on the probability distribution obtained from kernel density estimation, but also constrains the sample variation range through conditional sampling, dynamic boundaries, and truncation functions to preserve the continuity of the original trajectory in both the temporal and spatial dimensions.

In the temporal dimension, the first temporal sample is directly sampled from the estimated probability density distribution:

(58) $$x_{1}∼\hat{f}\left(x\right),$$

Subsequent temporal samples are generated from the conditional probability distribution based on the sample value of the previous moment:

(59) $$x_{t+1}∼\hat{f}\left(x_{t+1}|x_{t}\right),$$

where the conditional probability distribution considers the information of the previous time value $x_{t}$, thereby maintaining the continuity of the generated time series.

For the *j*-th sampled value $x_{kde}$ generated directly from the kernel density distribution, a truncation function is used to restrict it to the dynamic interval. The dynamic lower and upper bounds are defined as follows:

(60) $${\hat{x}}_{j}=\mathrm{clip}\left(x_{kde},x_{min},x_{max}\right),$$

(61) $$x_{min}=\max\left(x_{t-1}-\Delta_{max},x_{j}-2\Delta_{avg}\right),$$

(62) $$x_{max}=\min\left(x_{t-1}+\Delta_{max},x_{j}+2\Delta_{avg}\right).$$

Here, ${\hat{x}}_{j}$ represents the *j*-th constrained new sample, $x_{kde}$ represents the sampled value directly obtained from the Gaussian kernel density distribution, $x_{min}$ and $x_{max}$ represent the minimum and maximum constraint boundaries of the sampled value, respectively, $\Delta_{max}$ represents the maximum time interval, and $\Delta_{avg}$ represents the average time interval.

The spatial dimension adopts a sampling method consistent with the temporal dimension. First, initial coordinates are generated from the two-dimensional kernel density distribution corresponding to the optimal spatial candidate set; subsequently, subsequent coordinates are generated based on the conditional probability distribution of the previous coordinate to maintain the spatial correlation between trajectory points; finally, combined with the average variation range and maximum variation range of spatial coordinates, the dynamic boundaries are determined, and the range of sampled coordinates is controlled through a truncation function.

After completing the temporal sample and spatial coordinate sampling, the generated timestamps, longitudes, and latitudes are recombined into perturbed trajectory points, and a privacy-preserved trajectory dataset is formed in chronological order:

(63) $$T_{r}=\left\{d_{1},d_{2},\ldots ,d_{n}\right\},$$

The TTP sends the generated perturbed trajectory data to the LSP, and the LSP responds to the user’s continuous query requests based on the protected trajectory data and returns the location service results. In this way, STG-DPTP completes the processing workflow from original trajectory preprocessing, spatiotemporal hierarchical partitioning, probabilistic modeling and privacy selection, to constrained trajectory generation and publication.

## 3. Results and Discussion

### 3.1. Experimental Setup

#### 3.1.1. Experimental Environment and Dataset

The experimental environment was built on the Windows 11 operating system, and the QRDPP method and the comparison methods were implemented using the Python 3.12.13 programming language. The experimental data were obtained from the open-source GeoLife GPS trajectory dataset released by Microsoft Research Asia [43]. The dataset contains 17,621 real trajectory records collected from 182 users, with a total travel distance of more than 1.2 million kilometers and a total duration of more than 48,000 h. The trajectories span from April 2007 to August 2012 and consist of time-stamped GPS records with spatial coordinates, providing real-world spatiotemporal mobility characteristics for trajectory analysis.

Although the data collection period of GeoLife is relatively early, the purpose of this study is to evaluate the algorithmic privacy–utility characteristics of location and trajectory protection mechanisms rather than to characterize current mobility behavior. The dataset provides dense and real GPS trajectories with explicit spatial and temporal correlations, which are well suited to evaluating spatial query accuracy, trajectory reconstruction, and spatiotemporal privacy protection. Moreover, GeoLife continues to be adopted in recent trajectory privacy and differential privacy studies [44,45], which facilitates comparison with existing methods. Nevertheless, the use of a dataset collected during an earlier period may limit the evaluation of mobility patterns associated with more recent urban environments and mobile sensing technologies. Evaluation using more recent, heterogeneous, and large-scale trajectory datasets will therefore be considered in future work.

#### 3.1.2. Evaluation Metrics

Since QRDPP and STG-DPTP address different privacy-preserving scenarios, their evaluation metrics are selected according to their respective design objectives. For QRDPP, runtime is used to measure computational efficiency, while data utility is evaluated using relative error (RE) and logarithmic relative error (logRE). The privacy budget ϵ is treated as an experimental control parameter, where a smaller ϵ indicates stronger privacy protection and generally introduces larger perturbation.

The relative error RE and logarithmic relative error logRE are defined as

(64) $$RE=\frac{\left|N^{\prime }-N\right|}{\max(N,1)},$$

(65) $$logRE=\log_{10}\left(RE+\delta \right),$$

where δ is a small smoothing constant used to avoid the undefined logarithm when RE=0, *N* denotes the true count of the original location data within the query range, and $N^{\prime }$ denotes the corresponding count after privacy-preserving perturbation. A smaller logRE indicates higher query utility.

For STG-DPTP, Hausdorff distance, RMSE, AdvError, and time-series similarity are further adopted to evaluate trajectory shape preservation, reconstruction error, adversarial uncertainty, and temporal preservation, respectively. A smaller Hausdorff distance or RMSE indicates better spatial utility, a larger AdvError indicates greater uncertainty from the adversary’s perspective, and a time-series similarity closer to 1 indicates better temporal preservation. These complementary metrics jointly evaluate the main efficiency, utility, privacy, and spatiotemporal preservation objectives of the two proposed methods.

The baseline comparisons are descriptive because repeated trials are not available for every configuration. QRDPP uncertainty is evaluated using ten independent random-seed runs, whereas the STG-DPTP stability check uses ten trajectory-level bootstrap resamples of a fixed generated output; the latter is a sensitivity analysis rather than ten full algorithm executions or a pairwise significance test.

#### 3.1.3. Parameter Settings

The main experimental parameters of QRDPP and STG-DPTP are summarized in Table 1. The parameters were selected according to the structural characteristics of the two methods and were varied where necessary to evaluate their influence on privacy protection and data utility.

For QRDPP, the maximum tree depth, minimum subdivision count, and density threshold control adaptive spatial partitioning. The maximum depth prevents unrestricted recursion, $n_{\min}=2$ prevents subdivision when fewer than two points are available, and $\rho_{\mathrm{th}}=5$ stops relatively sparse branches. All three values are fixed throughout the reported comparisons; no density-threshold optimization experiment is claimed. The privacy budget shown in the results is the total budget ϵ, divided equally between the leaf and non-leaf pools. Different total budgets and query coverage ratios are examined descriptively to characterize the privacy–utility tradeoff and the influence of query scale. A smaller ϵ introduces larger Laplace noise, whereas a larger ϵ reduces the noise scale.

For STG-DPTP, the clustering parameters control the temporal and spatial granularity of trajectory modeling. A primary clustering value of $K_{t}=48$ provides an average temporal granularity of approximately 30 min over a 24-h period, while the alternative values are used to examine the influence of temporal clustering granularity. The secondary clustering values are varied from 5 to 60 to evaluate the effect of increasingly fine spatial partitioning. The privacy budget ϵ is varied to examine its effect on the privacy–utility tradeoff: smaller values impose stronger privacy constraints, whereas larger values increase the probability of selecting high-utility candidates and generally reduce reconstruction error. Uniform, Gaussian, and exponential adversarial priors are further considered to evaluate the stability of AdvError under different attacker assumptions.

In practical LBS deployments, the privacy budget should be selected according to the sensitivity of the requested service and its tolerance to utility loss. A smaller ϵ is more appropriate when location confidentiality is prioritized, whereas a larger ϵ may be adopted when query accuracy or trajectory utility is more critical. The values evaluated in this study are therefore intended to characterize the privacy–utility tradeoff rather than to prescribe a universal privacy budget for all LBS applications.

### 3.2. Experimental Results of QRDPP

The QRDPP results are analyzed descriptively from five aspects: data utility, query efficiency, the influence of spatial index structures, comparison with existing methods, and repeated-run scalability. Because QRDPP formally releases noisy node-count query answers rather than individual perturbed coordinates, its evaluation focuses on query error and computational performance.

#### 3.2.1. Effect of Dataset Size on Query Accuracy

**Figure 4:**
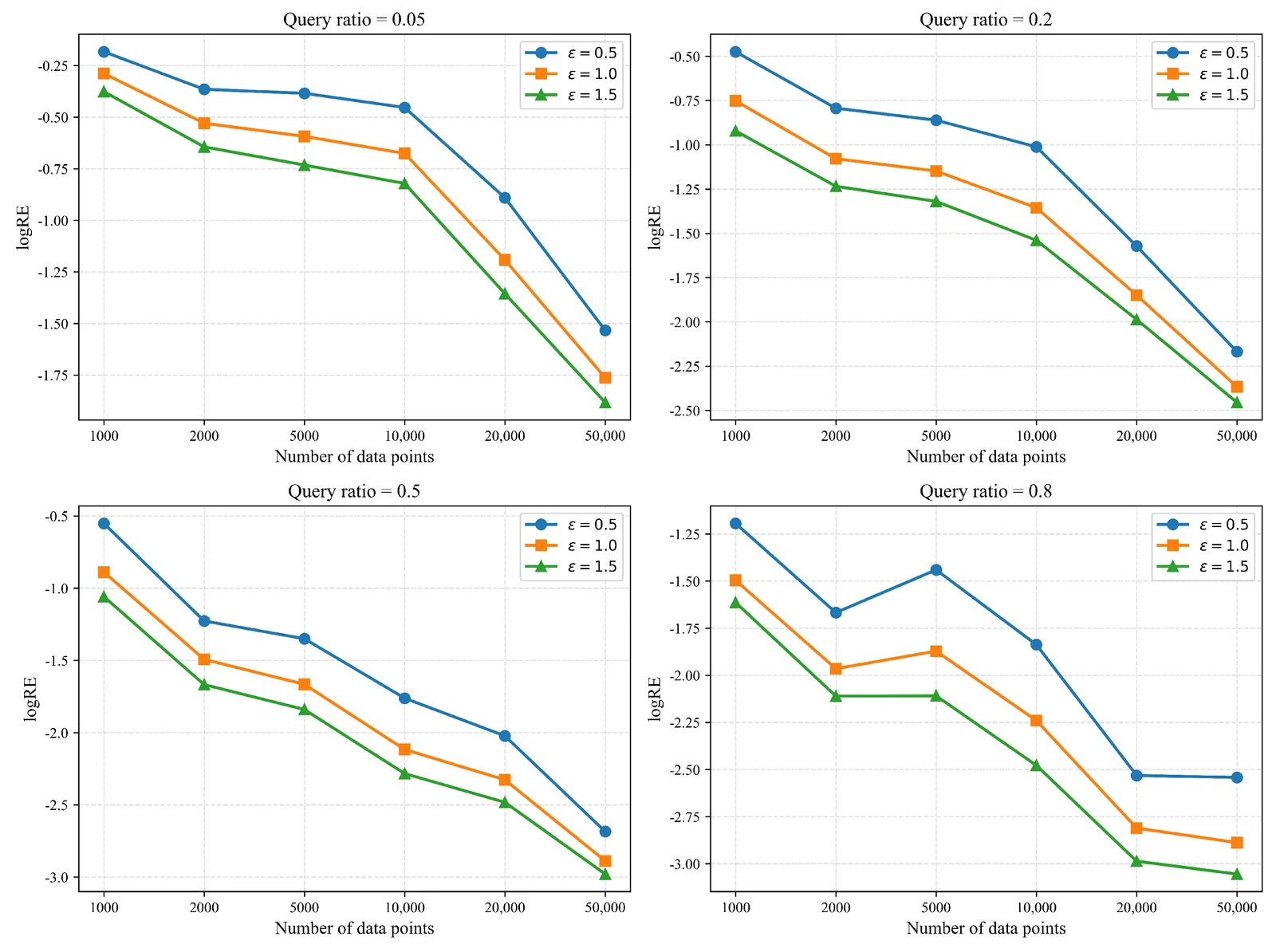
Effect of dataset size on logRE under different privacy budgets and query coverage ranges.

To analyze the influence of historical location data size on the data utility of QRDPP, location datasets with different sizes are selected, and the privacy budgets are set to ϵ=0.5,1.0,1.5. The logRE values are calculated under different query coverage ranges. The experimental results are shown in Figure 4.

As shown in Figure 4, under the same privacy budget, the logRE of QRDPP generally decreases as the dataset size increases, which indicates that increasing the dataset size helps improve the query accuracy of the published data. This is because a larger historical location dataset can provide more sufficient spatial distribution information, enabling the Q-R tree hybrid index to more accurately describe the spatial aggregation characteristics of the data and thus reduce the relative error in the query results. Meanwhile, under the same dataset size, a larger privacy budget leads to a lower logRE. This indicates that a larger privacy budget introduces less Laplace noise, making the perturbed results closer to the original query results.

In addition, the experimental results under different query coverage ranges also show that logRE generally decreases as the query range increases. This is because a larger query range contains a larger true count, so the relative contribution of Laplace noise in the aggregated node-count answer becomes smaller. Therefore, the deviation caused by differential privacy noise is diluted by the sample accumulation effect. As a result, QRDPP can show higher data utility under larger dataset sizes and larger query ranges.

#### 3.2.2. Effect of Dataset Size on Query Efficiency

**Figure 5:**
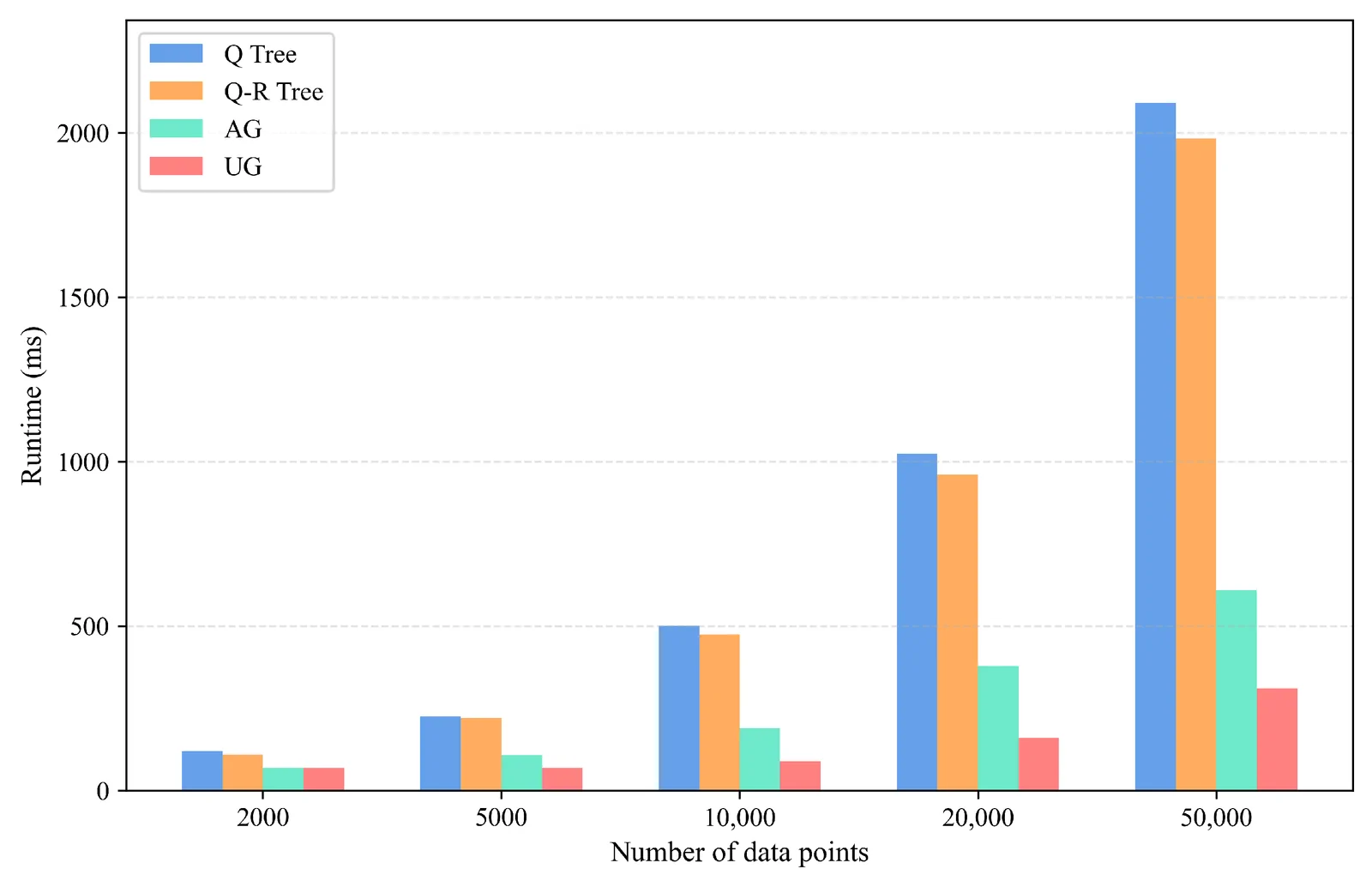
Runtime comparison of spatial index methods under different dataset sizes.

To verify the influence of historical location dataset size on the running efficiency of the algorithm, datasets with 2000, 5000, 10,000, 20,000, and 50,000 location points are selected. Under the condition that the query coverage range is 0.2, the uniform grid (UG), adaptive grid (AG), Q-tree, and the proposed Q-R tree hybrid spatial index structure are tested. Runtime measurements were repeated several times under each displayed setting, and the arithmetic mean is shown in Figure 5; these repeats support measurement averaging only and are not used for a formal significance test.

As shown in Figure 5, as the size of the historical location dataset continues to increase, the runtime of each method generally shows an upward trend. Compared with the traditional Q-tree index method, QRDPP adopts the Q-R tree hybrid index structure and sets the Q-tree stopping conditions based on heat density and subdivision level. This avoids continuously generating a large number of invalid child nodes in low-density regions and blank regions. Within the displayed measurements, its mean runtime is lower than that of the Q-tree method; this sentence describes the plotted values and does not assert statistical significance. Although UG and AG have shorter runtimes because they use simple grid structures, their spatial partitioning is relatively coarse and may split natural spatial clusters, resulting in reduced data utility. Thus, in the tested configurations, QRDPP provides lower measured runtime than the Q-tree baseline while retaining the utility pattern reported below.

The repeated-run analysis below extends the evaluation to 150,000 genuine points and additionally measures peak memory.

#### 3.2.3. Repeatability and Scalability Analysis

QRDPP was independently executed ten times with different random seeds at each dataset size using ϵ=1.0, a query coverage ratio of 0.20, and 200 random range queries per run. The tested sizes range from 2000 to 150,000 genuine GeoLife points without duplication or synthetic enlargement. We report the mean and sample standard deviation, the 95% confidence interval for runtime, and the peak working set measured in a clean process.

**Figure 6:**
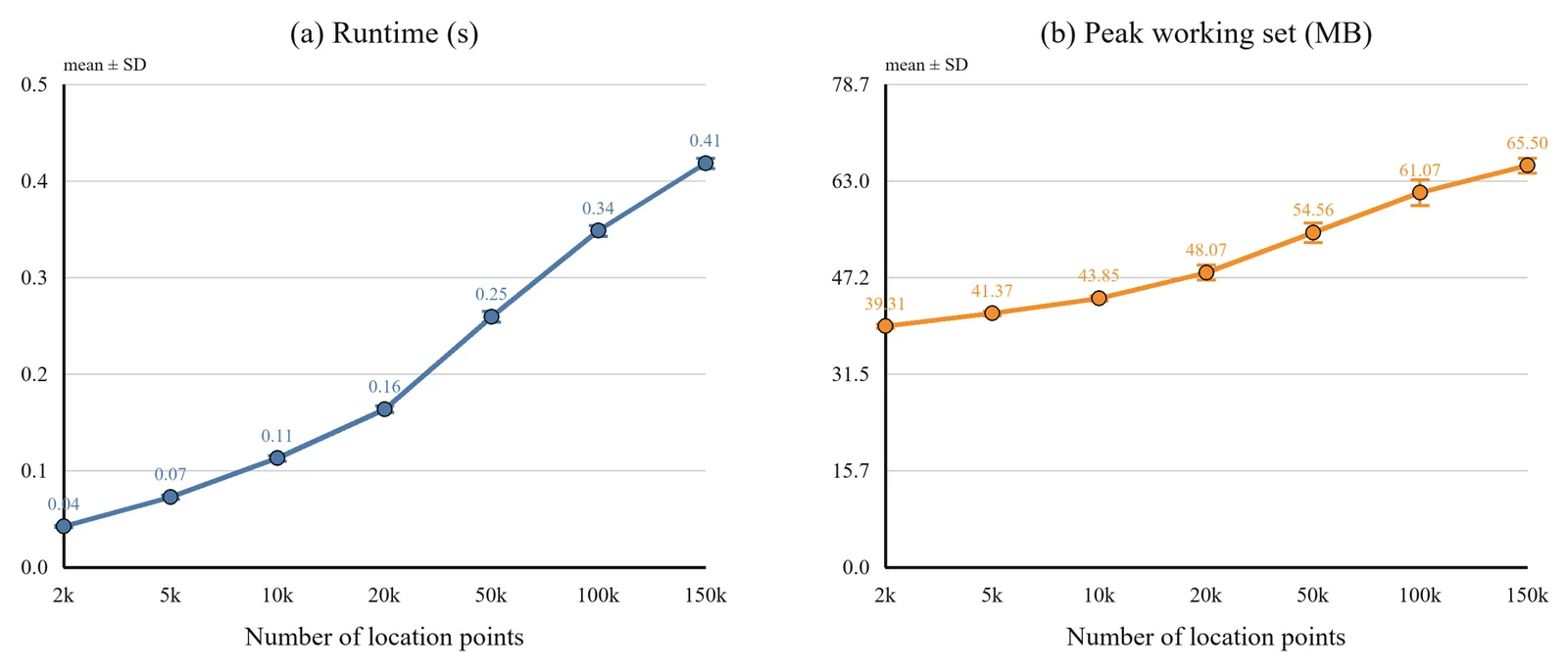
Repeatability and scalability of QRDPP over ten independent runs: (**a**) total runtime and (**b**) peak working-set memory under different numbers of genuine GeoLife location points. Error bars denote one sample standard deviation.

As shown in Figure 6 and Table 2, from 2000 to 150,000 points, mean runtime increased from 0.041±0.001 s to 0.406±0.005 s and peak memory from 39.3±0.3 MB to 65.5±1.2 MB; the mean logRE at 150,000 points was −2.5353±0.2171. The narrow runtime confidence intervals and gradual memory increase indicate stable execution within the tested range, while larger or highly concurrent workloads remain for future evaluation.

#### 3.2.4. Effect of Query Range and Spatial Index Structure on Data Utility

To further analyze the influence of different spatial index structures on query error in privacy protection scenarios, experiments are conducted under the conditions that the privacy budgets are ϵ=0.1 and ϵ=1.0, and the dataset size is 50,000. The query coverage ranges are set to 0.05, 0.2, 0.4, 0.6, and 0.8, and the logRE values of UG, AG, Q-tree, and QRDPP are compared. Since this part of the data mainly reflects the numerical differences under different query ranges, the original figure is converted into a table in this paper so as to more clearly present the specific results of different methods under different parameters, as shown in Table 3.

As shown in Table 3, as the query range ratio increases, the logRE of each method generally shows a decreasing trend, which indicates that a larger query range helps reduce the relative error. Under different query ranges and privacy budget conditions, the logRE of QRDPP is lower than that of UG, AG, and Q-tree, indicating that the proposed Q-R tree hybrid index structure can effectively improve data utility. The main reason for this difference is that UG and AG adopt relatively simple grid index structures, and the spatial partitioning granularity is limited. This may easily lead to uneven data point distribution in some regions, thereby affecting query accuracy. Although the Q-tree method has hierarchical spatial partitioning ability, if the stopping condition is not properly set, a large number of empty nodes may be generated when processing non-uniformly distributed data, which further affects query error. In contrast, QRDPP introduces the MBR indexing mechanism and combines it with the heat-density-based stopping condition, so it can organize spatial data more accurately and therefore obtain lower query relative error.

#### 3.2.5. Comparison with Existing Privacy Protection Methods

**Figure 7:**
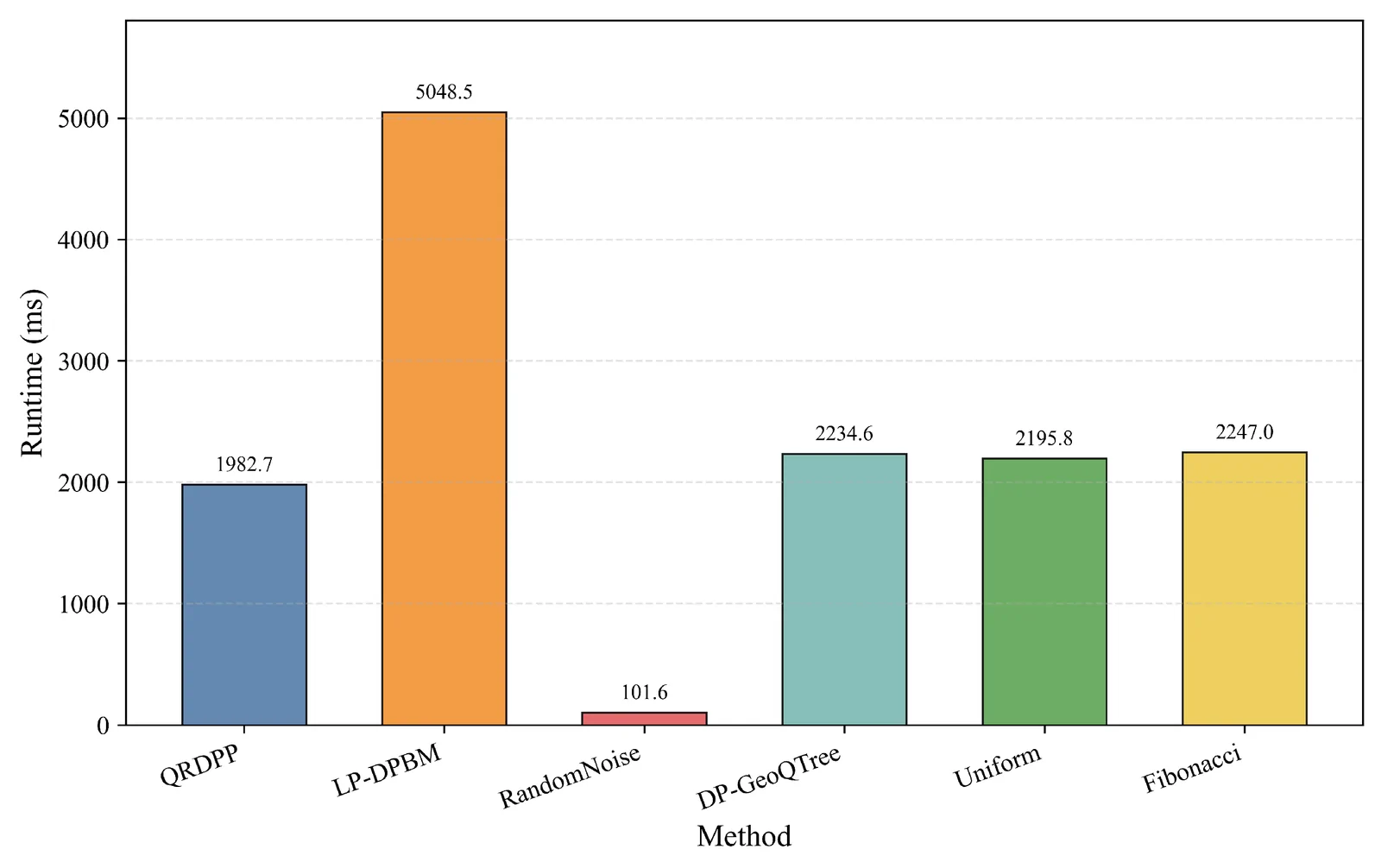
Runtime comparison of different privacy protection methods.

To verify the query efficiency of QRDPP in privacy protection scenarios, experiments are conducted under the conditions that the privacy budget is ϵ=1.0, the dataset size is 50,000, and the query coverage range is 0.2. QRDPP is compared with RandomNoise, DP-GeoQTree, Uniform, Fibonacci, and LP-DPBM in terms of runtime. The descriptive results are shown in Figure 7.

As shown in Figure 7, the runtime of QRDPP is higher than that of the RandomNoise method. This is because the RandomNoise method directly adds random noise to location points, and its processing procedure is relatively simple. In contrast, QRDPP needs to complete Q-R tree spatial partitioning and hierarchical privacy budget allocation before noise perturbation, which introduces certain additional computational overhead. In the displayed measurement, QRDPP has a lower runtime than LP-DPBM, DP-GeoQTree, Uniform, and Fibonacci. These are descriptive comparisons; without repeated baseline measurements and uncertainty estimates, no claim of statistical significance is made.

To further analyze the data utility of different methods, experiments are conducted under the conditions that the privacy budgets are ϵ=0.1,0.2,0.5,1.0,1.5,2.0, the dataset size is 50000, and the query coverage ranges are 0.2 and 0.8. The logRE values of QRDPP, RandomNoise, DP-GeoQTree, Uniform, Fibonacci, and LP-DPBM are compared. Since this part involves multiple privacy budgets and multiple methods, the original figure is converted into a table in this paper so as to more compactly show the specific results of different methods under different privacy budgets, as shown in Table 4.

As shown in Table 4, when the query range increases from 0.2 to 0.8, the logRE of each method generally shows a decreasing trend, which indicates that a larger query range can cover more original data samples and thus weaken the influence of differential privacy noise on the query results. Under all privacy budget settings, the logRE of QRDPP remains at a relatively low level, showing higher data utility. This is mainly due to the accurate partitioning of the data space by the Q-R tree hybrid index and the effective control of noise scale by the differentiated privacy budget allocation strategy. Because QRDPP releases noisy node-count answers rather than perturbed coordinates, its utility is interpreted through query error rather than coordinate preservation.

### 3.3. Experimental Results of STG-DPTP

To verify the effectiveness of the proposed trajectory privacy protection method based on spatiotemporal generalization and differential privacy, namely STG-DPTP, this paper further evaluates the experimental results across nine aspects: spatial trajectory similarity, temporal sequence preservation, dynamic bandwidth allocation, reconstruction error distribution, adversarial expected error, privacy budget influence, comparison with recent trajectory-synthesis baselines, bootstrap stability, and visualization of reconstructed trajectory points. Since continuous trajectory data simultaneously contain spatial coordinates and timestamp information, the evaluation of STG-DPTP focuses not only on whether the generated trajectory data can preserve the spatial distribution characteristics of the original trajectories, but also on whether the temporal correlation and trajectory utility can be maintained under privacy-preserving perturbation.

#### 3.3.1. Effect of Secondary Clustering Values on Spatial Trajectory Similarity

**Figure 8:**
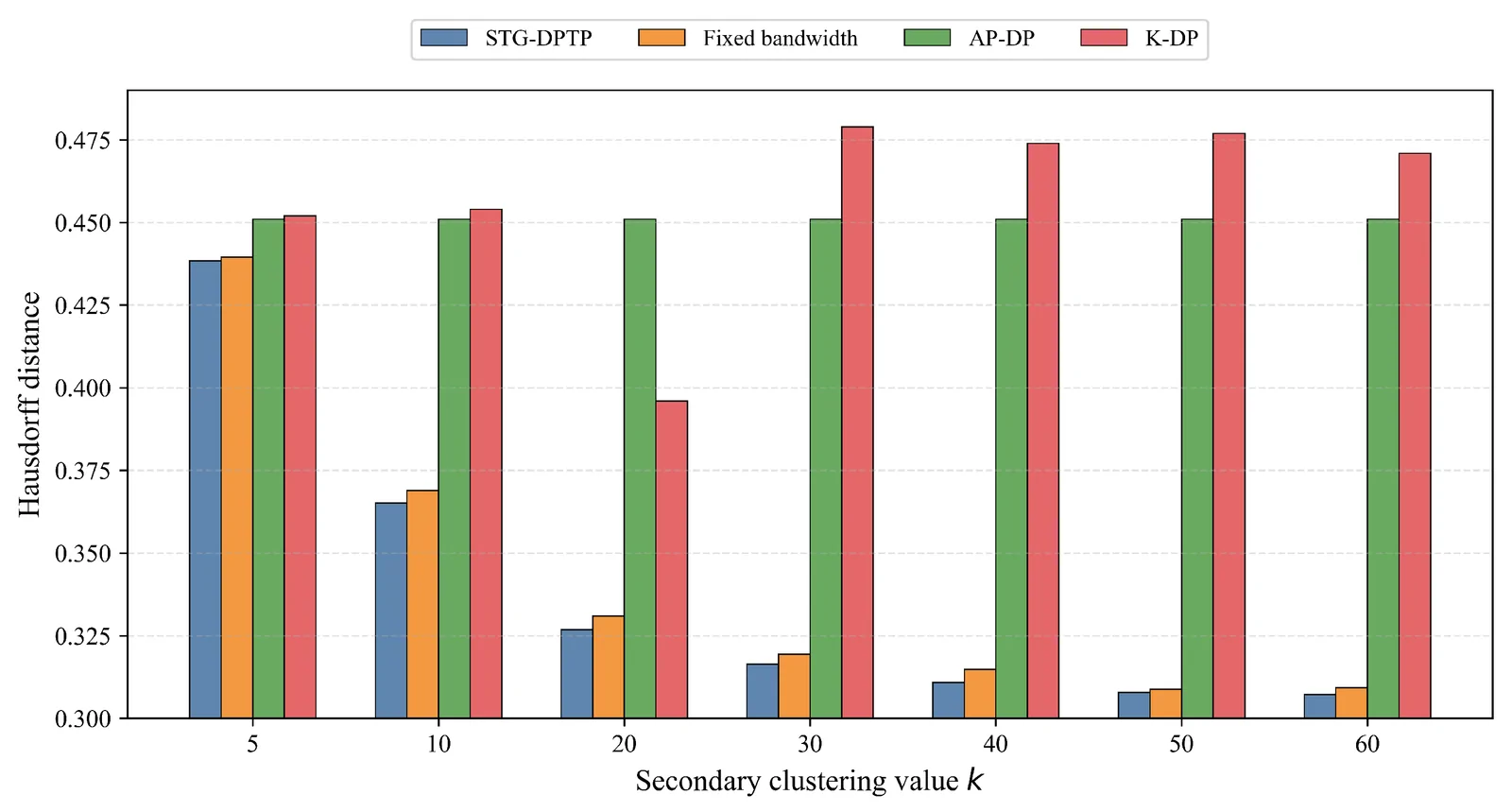
Hausdorff distance comparison under different secondary clustering values.

To verify the influence of secondary spatial clustering on trajectory shape preservation, this paper compares the Hausdorff distance of STG-DPTP, fixed bandwidth, AP-DP, and K-DP under different secondary clustering values. The secondary clustering values are set to k=5,10,20,30,40,50,60. The experimental results are shown in Figure 8.

As shown in Figure 8, the Hausdorff distance of the proposed STG-DPTP method gradually decreases as the secondary clustering value *k* increases. This is because a larger *k* enables the Bisecting K-means clustering method to divide the spatiotemporal characteristics of trajectory data more finely. With spatiotemporal fitting, dynamic parameter adjustment, and privacy-preserving selection by the exponential mechanism, the perturbed trajectories can more closely fit the original trajectory characteristics, thereby reducing the distance deviation. Compared with the fixed-bandwidth method, STG-DPTP can dynamically adjust the bandwidth according to the distribution characteristics of different clusters, and thus obtains lower Hausdorff distance under most parameter settings. In contrast, K-DP and AP-DP do not fully adapt to the internal characteristics of trajectory data. When the clustering value increases, the perturbation may further damage the trajectory structure, resulting in a larger trajectory shape deviation. Therefore, STG-DPTP shows better spatial trajectory similarity and stronger spatial distribution preservation ability.

#### 3.3.2. Effect of Clustering Values and Dynamic Bandwidth Allocation

To analyze the influence of clustering values on the temporal preservation and trajectory reconstruction quality of STG-DPTP, this paper evaluates the time-series similarity under different primary clustering values and the RMSE under different secondary clustering values. Since the two experiments mainly reflect numerical differences under different parameter settings, the original figures are converted into a combined table to present the results more compactly, as shown in Table 5.

As shown in Table 5a, the time-series similarity of STG-DPTP is higher than that of the fixed-bandwidth method under all primary clustering values. Meanwhile, as the primary clustering value increases, the temporal similarity of both methods generally improves, indicating that finer temporal clustering can more accurately describe the temporal distribution characteristics of trajectory data. Compared with fixed-bandwidth allocation, STG-DPTP dynamically adjusts the model parameters to adapt to the feature distribution of different clustering scales, so it can more accurately capture the similarity between time series and better preserve the inherent temporal correlation of trajectory data.

As shown in Table 5b, the RMSE values of both methods decrease as the secondary clustering value increases. This is because the increase in the number of secondary spatial clusters enables the trajectory data to be divided into more fine-grained spatial subregions, which helps the kernel density estimation model more accurately fit the local spatial distribution of trajectory points. Under all secondary clustering values, STG-DPTP obtains lower RMSE than the fixed-bandwidth method. This indicates that the dynamic bandwidth allocation strategy can better adapt to the spatial density differences among different clusters. Compared with fixed-bandwidth allocation, dynamic bandwidth allocation can reduce the sampling deviation caused by unsuitable bandwidth parameters, thereby improving the reconstruction quality of perturbed trajectories.

#### 3.3.3. Error Distribution Analysis of Reconstructed Trajectories

**Figure 9:**
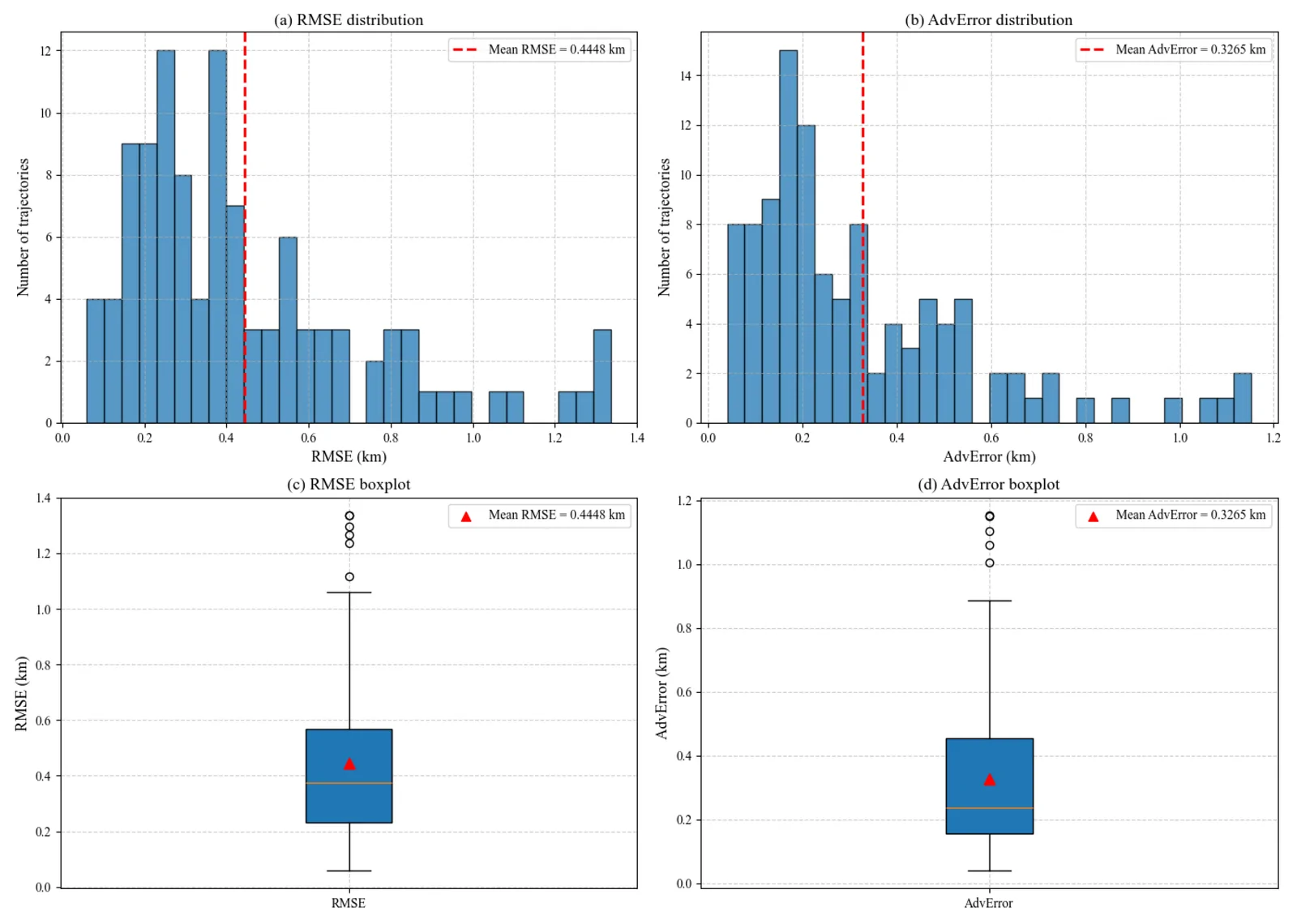
Error distribution and boxplot analysis of the reconstructed trajectories. (**a**) RMSE distribution; (**b**) AdvError distribution; (**c**) RMSE boxplot; (**d**) AdvError boxplot.

To further analyze the overall reconstruction error of the generated trajectory data, this paper calculates the RMSE and adversarial expected error (AdvError) between the original trajectories and the reconstructed trajectories. RMSE is used to evaluate the spatial deviation between the generated trajectories and the original trajectories, while AdvError reflects the expected spatial uncertainty from the adversary’s perspective. The error distribution and boxplot analysis are shown in Figure 9.

As shown in Figure 9, the RMSE values of most reconstructed trajectories are concentrated within a relatively low range, indicating that the generated trajectories retain stable spatial utility. In contrast, AdvError measures the expected localization uncertainty from the adversary’s perspective, and larger AdvError values indicate stronger privacy protection. Therefore, RMSE and AdvError should be interpreted jointly rather than in the same direction. The distributions and boxplots show that STG-DPTP maintains relatively low reconstruction errors while introducing a measurable level of adversarial uncertainty, reflecting the tradeoff between trajectory utility and privacy protection.

#### 3.3.4. Effect of Prior Distributions on AdvError

**Figure 10:**
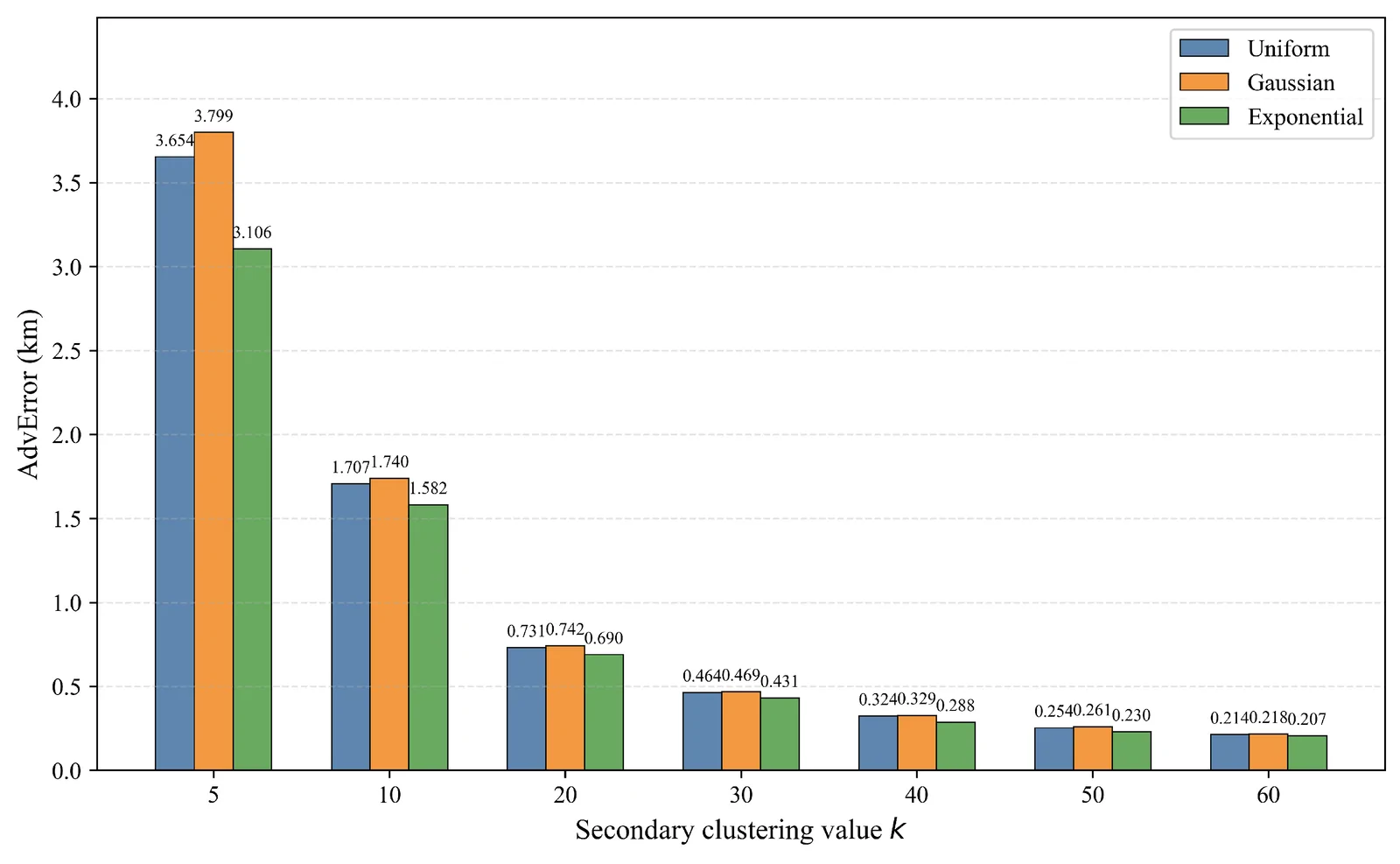
AdvError comparison under different prior distributions.

To analyze the influence of different adversarial prior distributions on privacy protection performance, this experiment compares the AdvError values under uniform, Gaussian, and exponential prior distributions. The secondary clustering values are set to k=5,10,20,30,40,50,60. The experimental results are shown in Figure 10.

As shown in Figure 10, the AdvError values under the three prior distributions generally decrease as the secondary clustering value *k* increases. This indicates that a finer secondary clustering granularity makes the generated trajectories closer to the original trajectory distribution, thereby improving the reconstruction quality of trajectory data. However, from the perspective of privacy protection, the decrease in AdvError also reflects the tradeoff between privacy protection strength and data utility. When the clustering granularity becomes finer, the generated trajectory can preserve more local spatial features, but the adversary’s expected localization error may also be reduced. Under different prior assumptions, the proposed method maintains a consistent variation trend, which indicates that STG-DPTP has stable performance under different background knowledge settings. This result further verifies that the proposed method can flexibly balance trajectory data utility and privacy protection under different adversarial assumptions.

#### 3.3.5. Comparative Analysis

**Figure 11:**
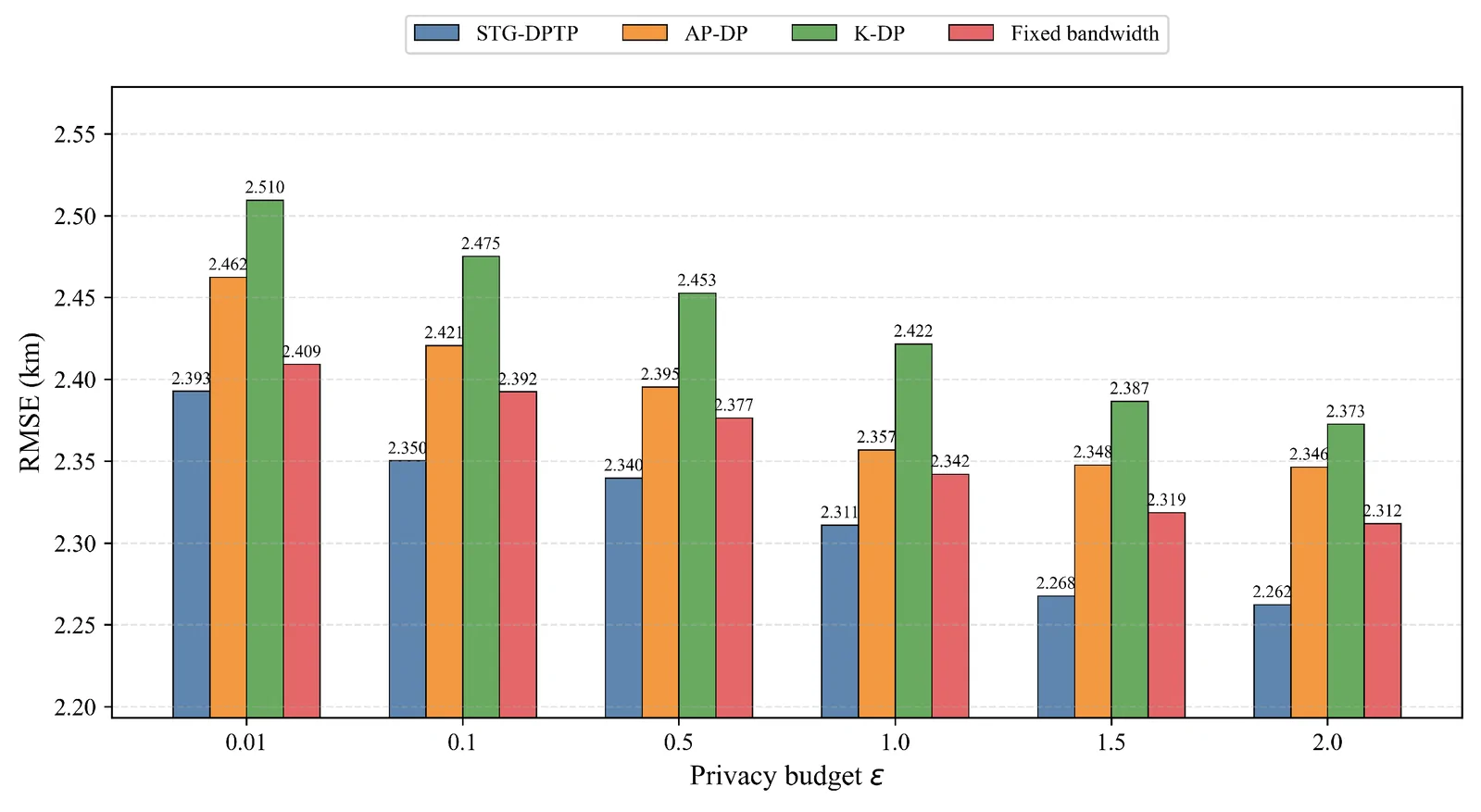
RMSE comparison under different privacy budgets.

To verify the advantage of the proposed method in balancing privacy protection and data utility, this experiment selects the privacy budgets as ϵ=0.01,0.1,0.5,1.0,1.5,2.0, and compares the RMSE of the proposed STG-DPTP method with AP-DP, K-DP, and the fixed-bandwidth method under different privacy budgets. The experimental results are shown in Figure 11.

As shown in Figure 11, the proposed STG-DPTP method has a relatively low RMSE under different privacy budgets, indicating that it has more advantages in balancing privacy protection and data utility. As the privacy budget ϵ increases, the RMSE of STG-DPTP shows an obvious downward trend and remains lower than that of the comparison methods. This means that when the privacy protection degree decreases, the proposed method can more effectively improve data utility and reduce trajectory perturbation error.

Compared with the fixed-bandwidth method, the proposed method has the ability of dynamic bandwidth adjustment, which enables it to better adapt to the distribution characteristics of trajectory data. Meanwhile, STG-DPTP fully considers the privacy leakage risk in the temporal dimension and provides targeted protection. In contrast, AP-DP and K-DP do not focus on this privacy risk, and their generalization mechanisms also have certain limitations, which lead to lower similarity between the data before and after generalization than that of the proposed method. In summary, the proposed STG-DPTP method performs better in both the comprehensiveness of privacy protection and the preservation of data utility.

#### 3.3.6. Comparison with PrivTrace and DP-STTS

To extend the comparison to recent trajectory-synthesis methods, we reproduced the open-source implementations of PrivTrace, which adaptively combines first- and second-order Markov models, and DP-STTS, which models transitions between spatiotemporal cubes [46,47]. The same GeoLife subset containing 109 source trajectories and 171,020 points was used, the total privacy budget was set to ϵ=1.0, and the random seed was fixed to 20260823. Because the methods output different numbers of sampled points, each trajectory was assigned equal total weight when calculating the dataset-level distributions and query answers.

**Figure 12:**
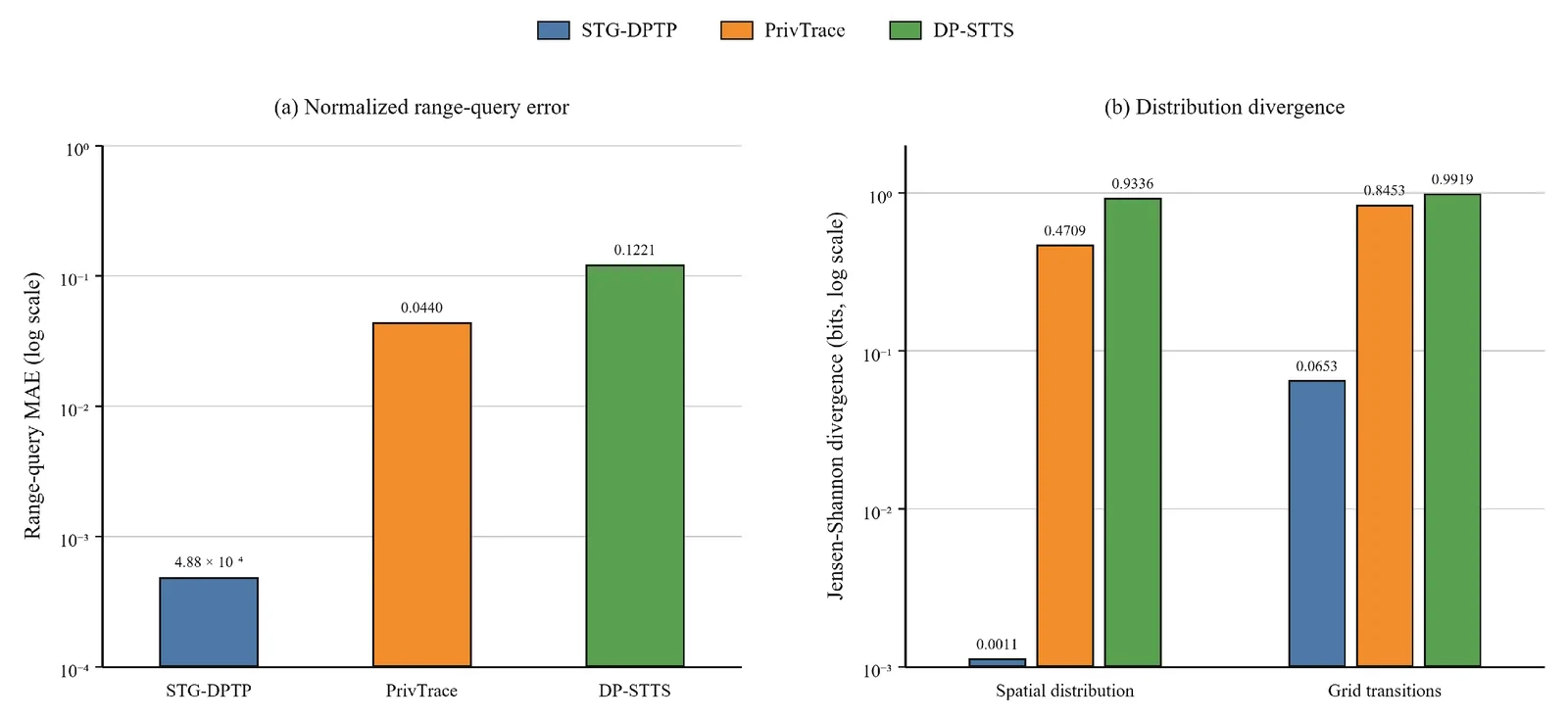
Fixed-seed pilot comparison with PrivTrace and DP-STTS at ϵ=1.0: (**a**) normalized range-query MAE; (**b**) spatial- and transition-distribution JSD. Both vertical axes use logarithmic scales.

Figure 12 reports the normalized mean absolute error (MAE) of 300 deterministic axis-aligned range queries and the Jensen–Shannon divergence (JSD) of the spatial and grid-transition distributions. Each query covers 0.20×0.20 of the normalized spatial domain, and both distribution metrics use a fixed 20×20 grid. Lower values indicate better preservation of the corresponding utility characteristic.

As shown in Figure 12a, STG-DPTP obtains a range-query MAE of 0.000488, compared with 0.044007 for PrivTrace and 0.122089 for DP-STTS, corresponding to descriptive reductions of 98.89% and 99.60%, respectively. Figure 12b shows the same pattern for distribution preservation: the spatial JSD values are 0.001128, 0.470881, and 0.933585, while the transition JSD values are 0.065349, 0.845345, and 0.991928 for STG-DPTP, PrivTrace, and DP-STTS, respectively. Within this tested setting, the lower errors of STG-DPTP are consistent with its fine-grained spatiotemporal clustering, adaptive density modeling, and constrained sampling, which retain local mobility mass and transition structure more closely.

These values constitute a single fixed-seed pilot reproduction rather than a repeated-run significance analysis. In addition, the neighboring-data definitions of the methods are not identical: the present STG-DPTP formulation uses point-level adjacency, whereas PrivTrace models trajectory records. Six source trajectories that crossed midnight were split into 115 within-day segments for DP-STTS because its released implementation assumes non-decreasing within-day time bins; no points were discarded. Therefore, the comparison should be interpreted as descriptive evidence of utility under the stated implementations and settings, rather than as proof of universally superior privacy–utility performance.

#### 3.3.7. Bootstrap Stability Analysis

To complement the fixed-seed comparison, we assessed the sensitivity of the STG-DPTP utility measurements using ten trajectory-level bootstrap resamples of the retained fixed generated output. The query sets were regenerated independently for the bootstrap replicates. This procedure measures variation caused by trajectory and query resampling; it does not represent ten independent executions of the complete multi-stage STG-DPTP generation pipeline and is not used for pairwise significance testing against the baseline methods.

Table 6 shows that the spatial-distribution JSD and normalized range-query MAE remained low across the resampled trajectory sets, while the top-20 hotspot Jaccard index averaged 0.707880. The wider variation in the transition JSD and hotspot overlap indicates that transition-level and hotspot-level summaries are more sensitive to the sampled trajectories than aggregate spatial mass and range-query answers. The results therefore provide a bounded stability check for the retained STG-DPTP output while preserving the distinction between bootstrap uncertainty and full algorithmic repeatability.

#### 3.3.8. Visualization Analysis of Spatiotemporal Fitting and Sampling Results

**Figure 13:**
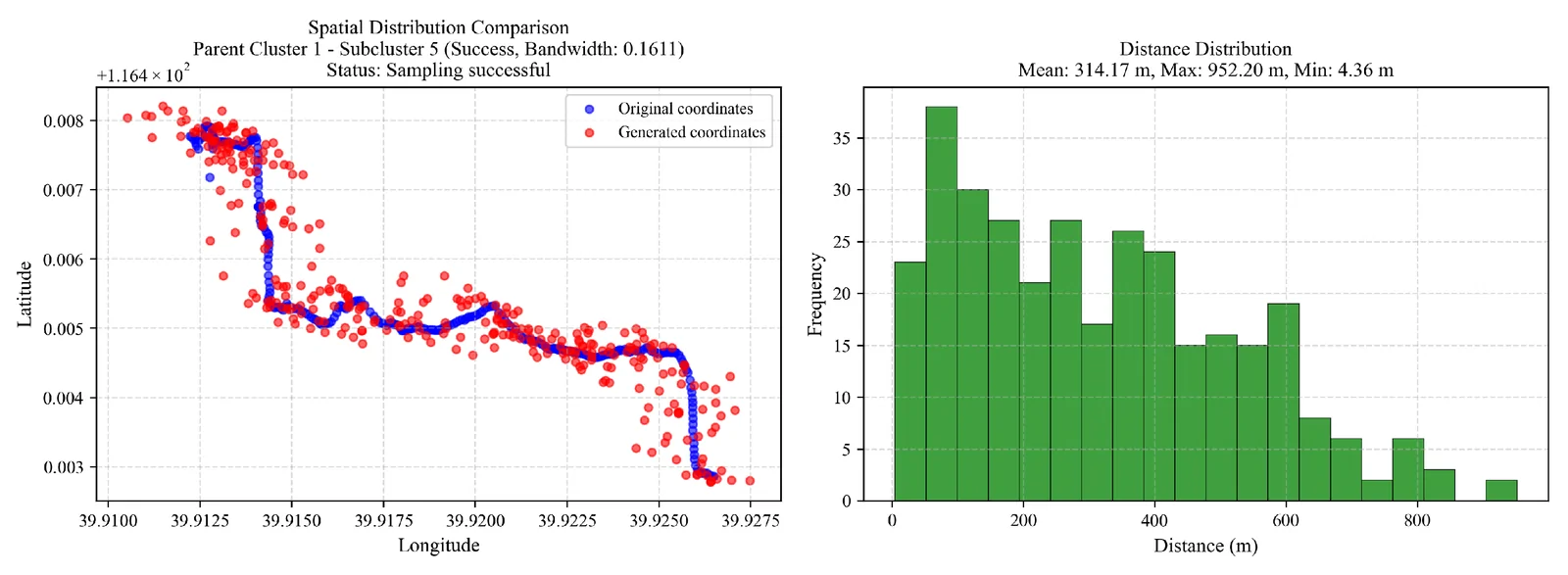
Spatial comparison before and after Gaussian kernel density estimation-based fitting and sampling.

To more intuitively show the differences between the fitted sampling data and the original data in both temporal and spatial dimensions, this experiment conducts visualization analysis from the perspective of spatiotemporal characteristics. Specifically, Figure 13 shows the comparison results before and after Gaussian kernel density estimation-based fitting and sampling in the spatial dimension, where the secondary clustering value is set to 48, corresponding to parent cluster 1 and subcluster 5. The scatter plot on the left clearly presents the distribution relationship between the original coordinates and the generated coordinates in the longitude–latitude space, while the distance distribution histogram on the right quantitatively shows the spatial distance difference between the generated coordinates and the original coordinates through distance frequency statistics.

**Figure 14:**
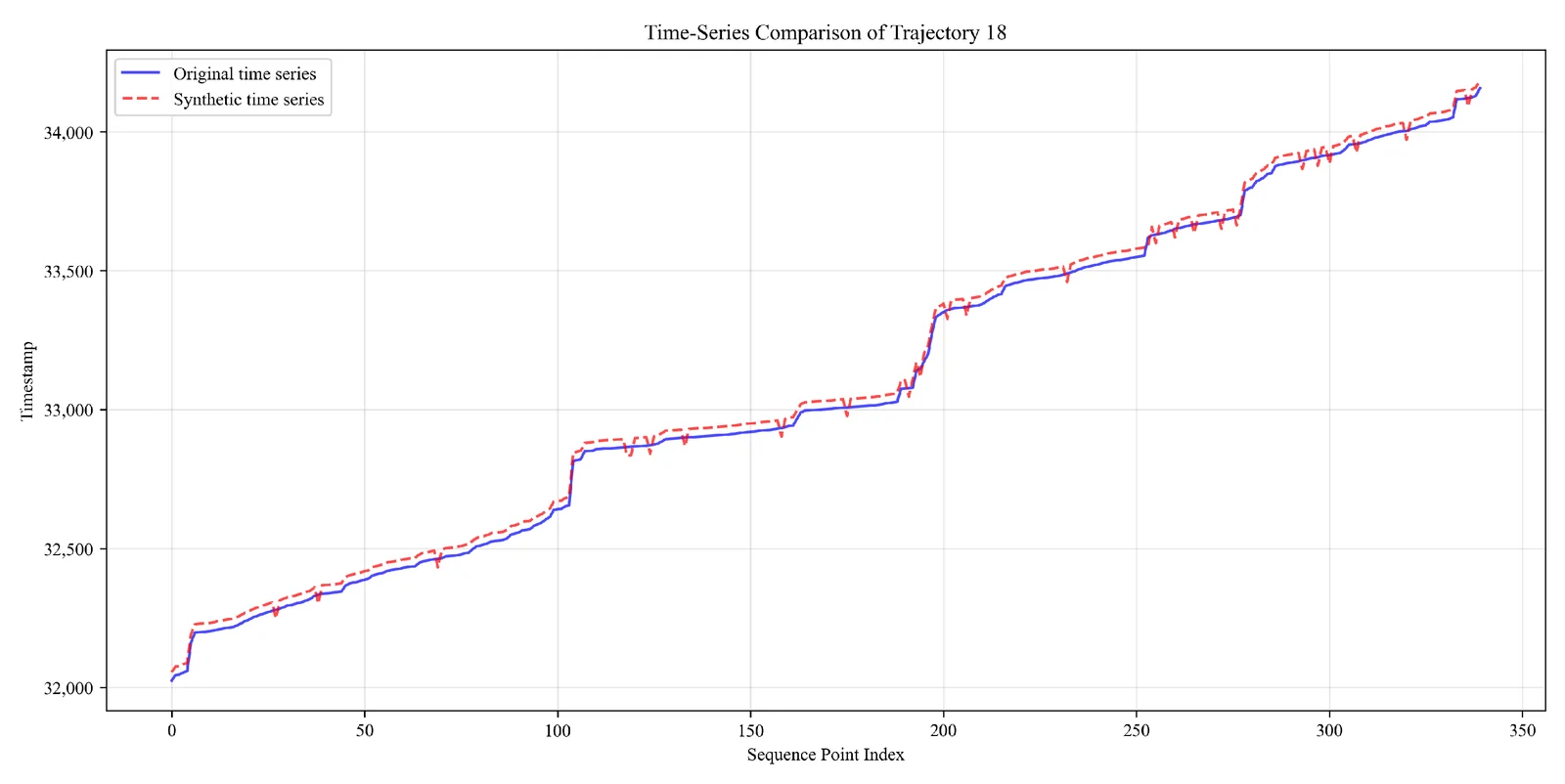
Time-series comparison before and after perturbation.

Figure 14 shows the comparison results of the time series before and after perturbation, where the primary clustering value is set to 48, corresponding to time series 18. The figure intuitively presents the trend consistency between the original time series and the synthetic time series, reflecting the fitting effect of the synthetic time series on the original time series in the temporal dimension.

The observed fitting performance benefits from the coordinated use of spatiotemporal feature modeling, dynamic parameter adaptation, and privacy-preserving candidate selection, which helps preserve the main spatial and temporal characteristics of the original data. However, the current experiments focus primarily on the privacy–utility behavior of STG-DPTP and do not include a dedicated large-scale runtime evaluation. Since hierarchical clustering, kernel density estimation, Bayesian optimization, and candidate selection introduce additional computational costs, its performance on substantially larger trajectory collections and high-frequency online queries remains to be further evaluated.

## 4. Conclusions

This paper studied the problem of privacy protection for location and trajectory data in location-based services (LBSs) and proposed two differential privacy-based methods for different query scenarios. For discrete query scenarios, QRDPP combines Q-R tree retrieval with differentiated privacy-budget allocation and applies the Laplace mechanism to hierarchical node-count statistics under the fixed-tree, record-level model stated in the Section 2. The total budget is divided between leaf and non-leaf count releases; QRDPP does not directly perturb or publish longitude–latitude coordinates. For continuous query scenarios, STG-DPTP was proposed by integrating spatiotemporal clustering, Gaussian kernel density estimation, dynamic bandwidth optimization, exponential mechanism-based selection, and constrained sampling to protect trajectory privacy in both spatial and temporal dimensions.

The descriptive results on the GeoLife dataset indicate favorable QRDPP query utility and runtime within the tested configurations and show that STG-DPTP preserves the main spatial and temporal trajectory characteristics while limiting reconstruction error. These observations are not presented as statistically significant or universally generalizable differences. Overall, the proposed methods provide effective solutions for balancing privacy protection and data utility in discrete location queries and continuous trajectory queries within the evaluated settings. The current evaluation does not fully cover very large-scale, highly concurrent, or real-time multi-user LBS environments. Future work will focus on adaptive privacy budget allocation, computationally efficient real-time trajectory protection, and evaluation using more recent and larger-scale mobility datasets.

The present guarantees assume that raw records and mechanism randomness remain inside a correctly operating central TTP. A coalition that observes only the same released output remains subject to the stated differential privacy bound, whereas distinct releases concerning the same privacy unit require explicit composition and budget accounting. Compromise of a TTP module with raw-data, unperturbed-state, or randomness access is outside this bound and remains a single-point-of-failure risk. Future work will therefore investigate distributed noise generation, secure aggregation, and multi-server designs that reduce the dependence on a single trusted curator.

## Figures and Tables

**Table 1:** Main parameter settings used in the experiments.

Method	Parameter	Setting
QRDPP	Maximum Q-tree depth $d_{\max}$	11
QRDPP	Density threshold $\rho_{\mathrm{th}}$	5
QRDPP	Minimum subdivision count $n_{\min}$	2
QRDPP	Sensitivity Δf	1
QRDPP	Privacy budget ϵ	Total budget: 0.1, 0.2, 0.5, 1.0, 1.5, 2.0; $\epsilon_{L}=\epsilon_{N}=\epsilon /2$
QRDPP	Query coverage ratio	0.05–0.80, depending on the experiment
STG-DPTP	Primary clustering value $K_{t}$	3, 12, 24, and 48; $K_{t}=48$ is used as the default setting
STG-DPTP	Secondary clustering value $K_{2}$	5, 10, 20, 30, 40, 50, and 60
STG-DPTP	Privacy budget ϵ	0.01, 0.1, 0.5, 1.0, 1.5, and 2.0
STG-DPTP	Adversarial prior	Uniform, Gaussian, and exponential

**Table 2:** Repeatability and scalability of QRDPP at ϵ=1.0 and a query coverage ratio of 0.20. Values are reported over ten independent random seeds.

Location Points	logRE (Mean ± SD)	Runtime (s; Mean ± SD)	Runtime 95% CI (s)	Peak Working Set (MB; Mean ± SD)
2000	−1.6687±0.2370	0.041±0.001	[0.040, 0.042]	39.3±0.3
5000	−1.4772±0.3550	0.070±0.002	[0.069, 0.072]	41.4±0.4
10,000	−1.5547±0.2032	0.110±0.003	[0.108, 0.112]	43.9±0.4
20,000	−1.7392±0.2468	0.159±0.003	[0.157, 0.161]	48.1±1.2
50,000	−2.1819±0.2534	0.252±0.005	[0.248, 0.255]	54.6±1.6
100,000	−2.4776±0.2182	0.338±0.005	[0.334, 0.342]	61.1±2.1
150,000	−2.5353±0.2171	0.406±0.005	[0.402, 0.409]	65.5±1.2

**Table 3:** logRE comparison of four spatial index methods under different query coverage ranges and privacy budgets.

ϵ	Query Ratio	UG	AG	Q-Tree	QRDPP
0.1	0.05	−0.620	−0.750	−0.860	−0.889771
	0.20	−1.185	−1.420	−1.510	−1.625992
	0.40	−1.410	−1.650	−1.820	−1.914724
	0.60	−1.520	−1.810	−2.130	−2.166037
	0.80	−1.540	−1.850	−2.090	−2.219634
1.0	0.05	−1.320	−1.600	−1.700	−1.727716
	0.20	−1.680	−1.980	−2.280	−2.331064
	0.40	−1.820	−2.300	−2.500	−2.728988
	0.60	−2.030	−2.450	−2.830	−2.975500
	0.80	−2.356	−2.700	−2.950	−3.052670

**Table 4:** logRE comparison of different privacy protection methods under different privacy budgets and query coverage ranges.

Query Ratio	ϵ	QRDPP	DP-GeoQTree	LP-DPBM	RandomNoise	Uniform	Fibonacci
0.2	0.1	−1.610	−1.590	−1.530	−1.210	−1.420	−1.510
	0.2	−1.870	−1.805	−1.770	−1.442	−1.569	−1.738
	0.5	−2.180	−2.140	−2.080	−1.476	−1.760	−1.990
	1.0	−2.290	−2.230	−2.200	−1.508	−1.960	−2.100
	1.5	−2.430	−2.354	−2.330	−1.607	−2.200	−2.300
	2.0	−2.489	−2.354	−2.327	−1.630	−2.225	−2.293
0.8	0.1	−1.590	−1.580	−1.547	−1.210	−1.417	−1.508
	0.2	−1.877	−1.844	−1.785	−1.480	−1.615	−1.750
	0.5	−2.180	−2.140	−2.100	−1.507	−1.840	−2.060
	1.0	−2.330	−2.289	−2.220	−1.508	−1.970	−2.110
	1.5	−2.410	−2.360	−2.310	−1.607	−2.220	−2.270
	2.0	−2.433	−2.397	−2.325	−1.630	−2.225	−2.280

**Table 5:** Effect of clustering values on temporal similarity and RMSE.

**(a) Time-series similarity under different primary clustering values**			
Primary clustering value *k*	STG-DPTP	Fixed bandwidth	Improvement
3	0.9839	0.9782	0.0057
12	0.9862	0.9811	0.0051
24	0.9895	0.9825	0.0070
48	0.9917	0.9887	0.0030
**(b) RMSE under different secondary clustering values**			
Secondary clustering value *k*	STG-DPTP	Fixed bandwidth	RMSE reduction
5	4.743585	4.953585	0.210000
10	2.300091	2.480091	0.180000
20	1.018025	1.118025	0.100000
30	0.646840	0.716840	0.070000
40	0.447948	0.477948	0.030000
50	0.348174	0.350174	0.002000
60	0.296335	0.297435	0.001100

**Table 6:** Trajectory-level bootstrap sensitivity of STG-DPTP. Values are based on ten bootstrap resamples of the retained fixed generated output; CI denotes a two-sided 95% confidence interval.

Metric	Mean	Sample SD	95% CI
Spatial-distribution JSD	0.028722	0.012448	[0.019817, 0.037627]
Normalized range-query MAE	0.004348	0.001116	[0.003550, 0.005146]
Grid-transition JSD	0.146859	0.017396	[0.134414, 0.159303]
Top-20 hotspot Jaccard index	0.707880	0.105037	[0.632741, 0.783019]

## Data Availability

The data supporting the findings of this study are available from the corresponding author upon reasonable request.
